# Mouse and human share conserved transcriptional programs for interneuron development

**DOI:** 10.1126/science.abj6641

**Published:** 2021-12-10

**Authors:** Yingchao Shi, Mengdi Wang, Da Mi, Tian Lu, Bosong Wang, Hao Dong, Suijuan Zhong, Youqiao Chen, Le Sun, Xin Zhou, Qiang Ma, Zeyuan Liu, Wei Wang, Junjing Zhang, Qian Wu, Oscar Marín, Xiaoqun Wang

**Affiliations:** 1 State Key Laboratory of Brain and Cognitive Science, CAS Center for Excellence in Brain Science and Intelligence Technology (Shanghai), Institute of Biophysics, Chinese Academy of Sciences, Beijing, 100101, China; 2 University of Chinese Academy of Sciences, Beijing 100049, China; 3 IDG/McGovern Institute for Brain Research, Tsinghua-Peking Center for Life Sciences, School of Life Sciences, Tsinghua University, Beijing 100084, China; 4 Centre for Developmental Neurobiology, Institute of Psychiatry, Psychology and Neuroscience, King’s College London, London SE1 1UL, United Kingdom; 5 MRC Centre for Neurodevelopmental Disorders, King’s College London, London SE1 1UL, United Kingdom; 6 State Key Laboratory of Cognitive Neuroscience and Learning, Beijing Normal University, Beijing, 100875, China; 7 IDG/McGovern Institute for Brain Research, Beijing Normal University, Beijing, 100875, China; 8 Beijing Institute of Brain Disorders, Laboratory of Brain Disorders, Ministry of Science and Technology, Collaborative Innovation Center for Brain Disorders, Capital Medical University, Beijing, 100069, China

## Abstract

Genetic variation confers susceptibility to neurodevelopmental disorders by affecting the development of specific cell types. Changes in cortical and striatal ɣ-aminobutyric acid-expressing (GABAergic) neurons are common in autism and schizophrenia. Here we used single-cell RNA sequencing to characterize the emergence of cell diversity in the human ganglionic eminences, the transitory structures of the human fetal brain where striatal and cortical GABAergic neurons are generated. We identified regional and temporal diversity among progenitor cells underlying the generation of a variety of projection neurons and interneurons. We found that these cells are specified within the human ganglionic eminences by transcriptional programs similar to those previously identified in rodents. Our findings reveal an evolutionarily conserved regulatory logic controlling the specification, migration, and differentiation of GABAergic neurons in the human telencephalon.

The general organization and cellular architecture of the telencephalon are conserved among mammals, but its size and complexity vary enormously between rodents and primates. Between mouse and human, the cerebral cortex differs 1,000-fold in size (1, 2) and varies in the types, proportions, and distributions of cells (3–5). Global transcriptomic analyses revealed differences in gene expression patterns between mouse and human (6–10), whereas single-cell transcriptomic analyses found conservation in the cellular composition of the cerebral cortex. Most cell types found in rodents, monkeys, and humans are homologous (11, 12). This raises the question of whether the unique features of the human telencephalon arise through fundamental changes in the gene regulatory networks controlling the development of this brain structure or via alternative mechanisms.

The cerebral cortex contains two main classes of neuron which derive from distinct structures in the developing telencephalon. Excitatory cortical neurons originate from progenitor cells in the developing pallium, whereas GABAergic neurons are generated in the ganglionic eminences, the transitory structures of the fetal brain which also give rise to the basal ganglia (13, 14). Although we have made substantial progress in elucidating the development of excitatory neurons in the human cortex (14, 15), our understanding of the generation of GABAergic neurons in the medial, lateral, and caudal ganglionic eminences (MGE, LGE and CGE, respectively) is very limited (16, 17). For instance, single-cell transcriptomic studies have identified the molecular signatures of glutamatergic lineages in the developing cortex (18–23), but similar insights into the development of the human ganglionic eminences remain fragmentary (10, 24). Here, we investigated the transcriptional trajectories of cells in the developing human ganglionic eminences and found conservation in the genetic programs controlling the development of GABAergic neurons in mice and humans. Our study offers insights into the molecular regulation of neurogenesis and the mechanisms underlying the diversification of GABAergic neurons.

## Cell diversity in the human ganglionic eminences

**Fig. 1:**
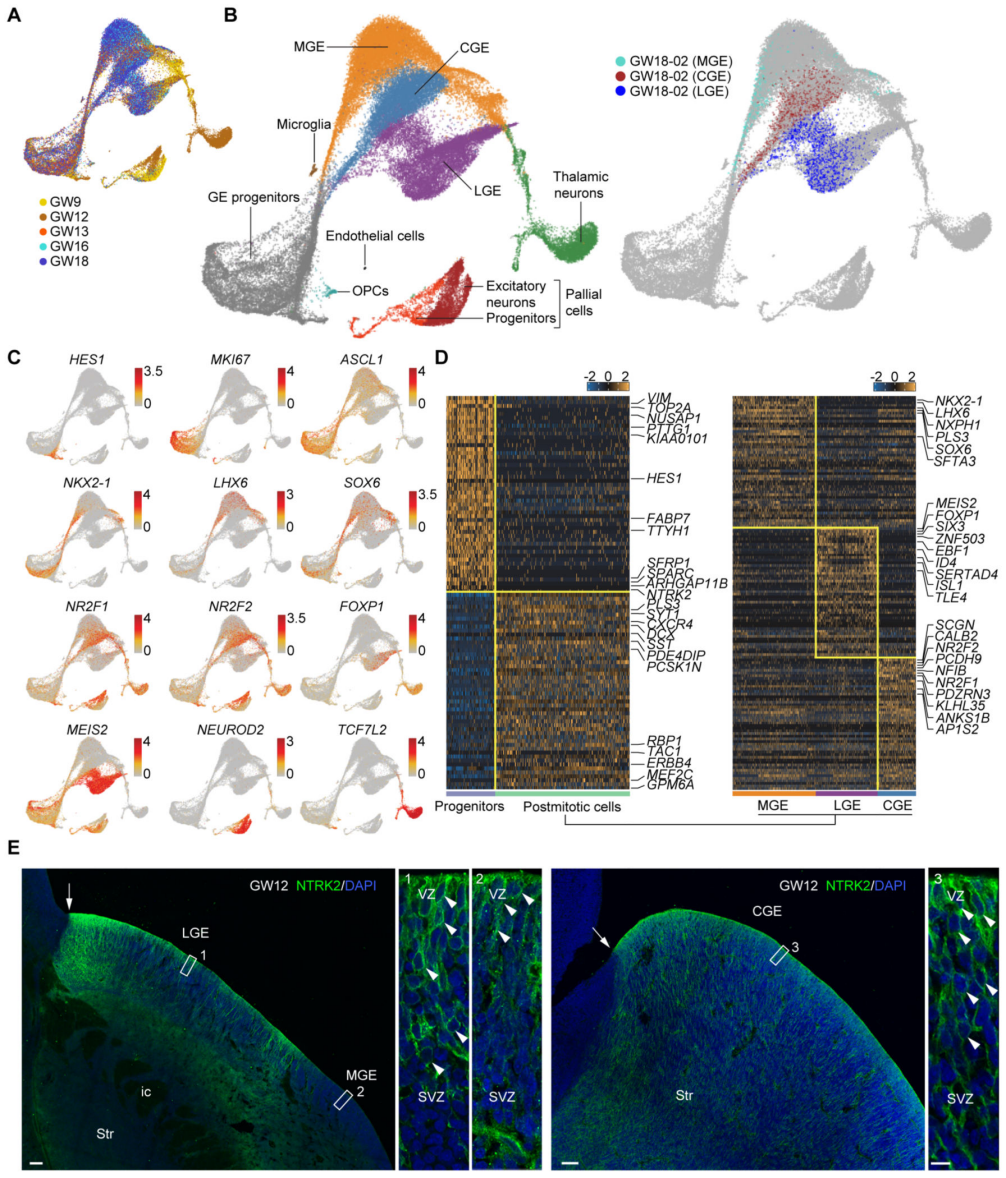
Major sources of transcriptional heterogeneity among cells from the human ganglionic eminences. (**A**) Clustering of individual cells from different gestational weeks by UMAP. (**B**) Annotation of cell clusters based on gene expression. The schema on the right illustrates the distribution of cells obtained from individual dissections of the MGE, LGE and CGE in relation to the unsupervised clustering of all cells. (**C**) Gene expression visualized by UMAP. Each dot represents an individual cell colored according to the expression level (red, high; grey, low). (**D**) Heatmaps illustrating differentially expressed genes (DEGs) enriched in progenitors and post-mitotic cells in the ganglionic eminences (left) and among post-mitotic cells in the MGE, LGE and CGE (right). (**E**) Expression of NTRK2 in progenitor cells in the ganglionic eminences at GW12. The areas in white boxes are shown at high magnification. Scale bars, 200 μm (LGE/MGE), 100 μm (CGE), 10 μm (boxes 1-3).

We used a droplet-based platform to study the transcriptomic profile of individual cells in the developing human ganglionic eminences. To this end, we dissected the ganglionic eminences across gestational weeks (GW) 9-18 (Table S1), which overlap with the peak of neurogenesis in this region, and performed single-cell RNA sequencing (scRNA-seq). Following quality control (fig. S1A), the transcriptional profiles of 56,412 single cells across GW9-18 were obtained and analyzed collectively using unsupervised clustering (Fig. 1A and fig. S1B). Unsupervised clustering of cellular transcriptional identities by uniform manifold approximation and projection (UMAP) dimensionality reduction revealed the existence of 10 cell clusters (Fig. 1B and Table S2), which we annotated using well-known cell type-specific markers (Fig. 1C and fig. S1C). The four main clusters correspond to progenitor cells and post-mitotic cells from the MGE, LGE and CGE (Fig. 1B). In addition, we identified smaller cell clusters containing oligodendrocyte progenitor cells (OPCs), microglia, endothelial cells, thalamic precursors, and pallial cells (Fig. 1B). The last two groups derive from tissues adjacent to the ganglionic eminences that were included in the dissection in the smallest samples (Fig. 1A). We used independent samples from two different stages to confirm data repeatability (fig. S1E) and validated the accuracy of unsupervised clustering using a sample in which the MGE, LGE and CGE were manually dissected and analyzed independently (Fig. 1B). We also performed differential gene expression analysis to detect the genes that best distinguish dividing progenitors from postmitotic cells in the ganglionic eminences, as well as postmitotic cells from the MGE, LGE and CGE (Fig. 1D, fig. S1D, and Tables S3 and S4). Apart from known markers, we identified *NTRK2*, which encodes the BDNF and NT-4 receptor (25), as a marker of progenitor cells in the human ganglionic eminences (Fig. 1D). Immunohistochemistry staining confirmed that NTRK2 expression is strong in the progenitor domains of the LGE and CGE (Fig. 1E). We also identified genes that distinguish among postmitotic cells with MGE, LGE and CGE identity, including multiple previously reported regional markers (Fig. 1D, fig. S1D and Table S3), as well as a small population of putative GABAergic neurons among thalamic cells (fig. S1F). Thus, the analysis of cellular transcriptomes revealed regional identities in the developing human ganglionic eminences along with some molecular signatures for specific cell types.

### Conserved genetic regulation of progenitor cells

**Fig. 2:**
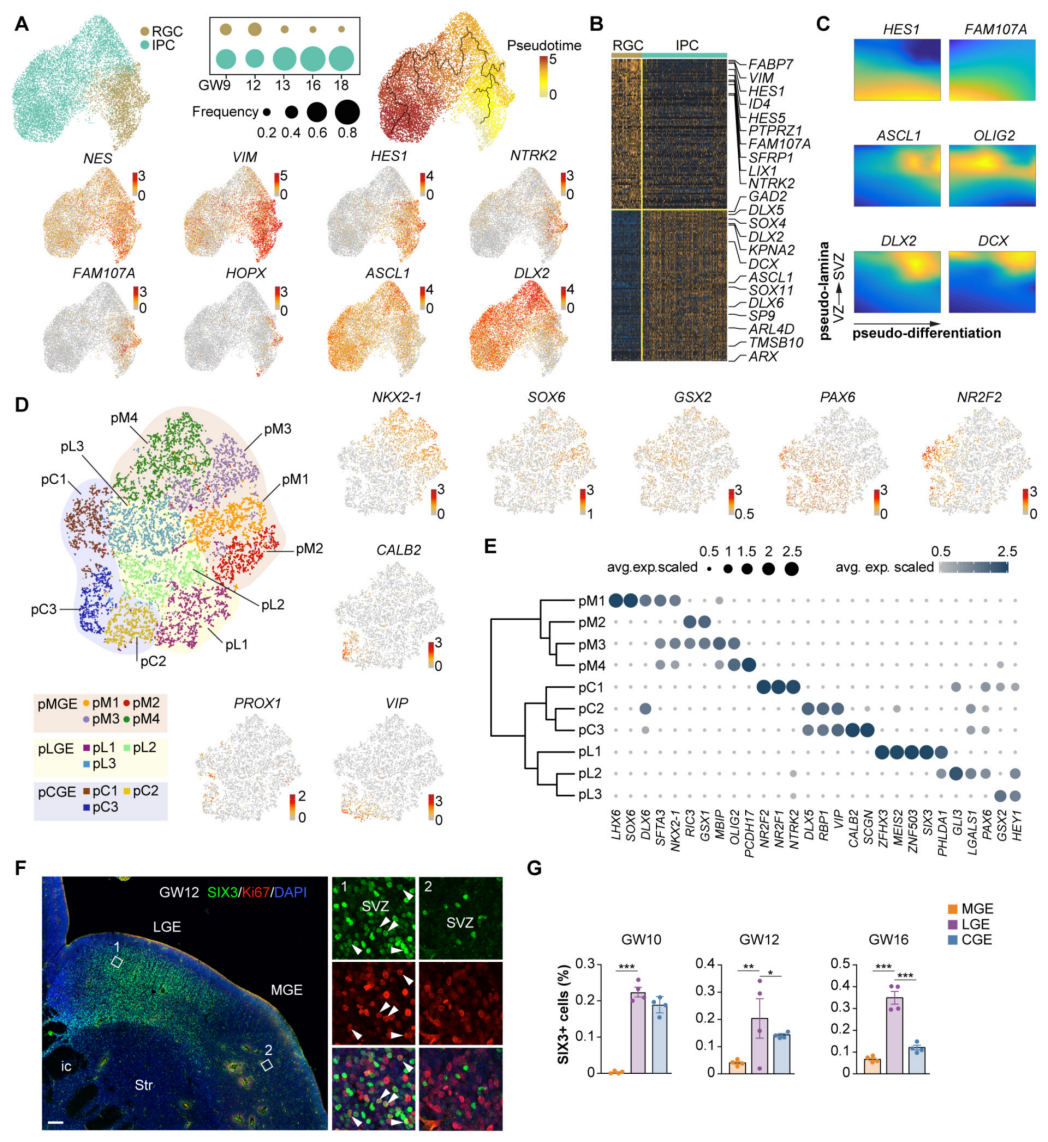
Cell diversity and genetic regulation of progenitor cells in the human ganglionic eminences. (**A**) Clustering of individual progenitor cells in the human ganglionic eminences (top left) and gene expression visualized by UMAP. Each dot represents an individual cell colored according to the expression level (red, high; grey, low). The developmental trajectory of progenitor cells is analyzed via pseudotime alignment (top right). The inset illustrates the ratio of RGCs and IPCs among ganglionic eminence progenitor cells through GW9-18. (**B**) Heatmap illustrating DEGs that distinguish between RGCs and IPCs in the human ganglionic eminences. (**C**) Gene expression patterns along pseudo-differentiation (x-axis) and pseudo-lamina (y-axis) coordinates. (**D**) Cell diversity among progenitor cells in the human ganglionic eminences visualized by t-SNE. The regional identity of progenitor cells was established according to their characteristic patterns of gene expression. (**E**) Differentially expressed genes among progenitor cells. The relationships among subclusters of GE progenitors is illustrated via dendrogram. The size of the dots and the color bar were scaled with the average expression of the corresponding genes. (**F**) Expression of SIX3 and Ki67 in the human LGE and MGE at GW12. The areas in white boxes are shown at high magnification. Scale bars, 200 μm (left), 20 μm (right). (**G**) Quantification of the cell ratio of SIX3+ cells in LGE, MGE and CGE cells at GW10, GW12 and GW16, respectively. Data are presented as means ± s.e.m. (*n* = 4 samples from 3 individual experiments, * *p* < 0.05, ** *p* < 0.01, *** *p* < 0.001, one-way ANOVA).

Neural progenitor cells in the ganglionic eminences (GE progenitors in Fig. 1B) include radial glial cells (RGCs) with neural stem characteristics and intermediate progenitor cells (IPCs), which derived from RGCs and are committed towards the neuronal lineage (16). We employed a list of established markers to delineate both types of progenitor cells in the human ganglionic eminences and visualized their developmental trajectories using pseudotime alignment (Fig. 2A). We then carried out differential gene expression analysis to unbiasedly identify genes whose expression best distinguish RGCs and IPCs in the human ganglionic eminences (Fig. 2B, fig. S2A and Table S5). In addition to genes that are expressed in progenitor cells in the mouse ganglionic eminences, such as *NES, VIM, HES1* and *ASCL1*, we also found other genes characteristically enriched in progenitor cells. For example, RGCs express *NTRK2* and *FAM107A*, while IPCs express *TMSB10* and *KPNA2* (Fig. 2, A and B).

Progenitor cells in the human ganglionic eminences are spatially organized in two adjacent niches, a relatively thin ventricular zone (VZ) and a large subventricular zone (SVZ) (figs. S2, B–D). In mice, RGCs and IPCs segregate between the VZ and SVZ, respectively. Analysis of gene expression trajectories across pseudo-lamina and pseudo-differentiation axes revealed a similar distribution for RGCs and IPCs in the human ganglionic eminences (Fig. 2C). This organization contrasts with the developing pallium, in which RGCs expressing *FAM107A* and *HOPX* are the most abundant type of progenitor cells in the SVZ (26–28). We found that most progenitor cells expressing FAM107A are in the VZ of the human ganglionic eminences (Fig. 2C and fig. S2C). Although we also detect a small population of RGCs that co-express *FAM107A* and *HOPX* (Fig. 2A), most progenitor cells in the human ganglionic eminences have the transcriptional signature of IPCs throughout the peak stages of neurogenesis (Fig. 2A).

We used unsupervised clustering to classify progenitor cells in the human ganglionic eminences into 10 transcriptionally unique clusters with unique gene expression profiles (Fig. 2D and fig. S3A). We observed that these progenitor clusters could be readily segregated according to their regional identity using the expression of genes involved in the patterning of the ganglionic eminences in mice (29). For example, expression of *NKX2-1* and *SOX6* characterized progenitor cells in the MGE (Fig. 2, D and E, fig. S3B and Table S6). Expression of *PAX6* was common to progenitor cells in the LGE and CGE, but CGE progenitors were further characterized by the expression of *PROX1* and *NR2F2* (Fig. 2, D and E, fig. S2D and fig. S3B). Further analysis identified *SIX3* as a gene differentially expressed among LGE progenitors (Fig. 2E). We validated this later finding using immunohistochemistry and confirmed that SIX3 is highly enriched among dividing progenitor cells in the SVZ of the human LGE from GW10-16 (Fig. 2, F and G). Since *Six3* plays a role in the generation of striatal medium spiny neuron which originate from LGE progenitors in mice (30), our results reinforce the notion that eminence-specific genetic programs similar to those described in rodents seem to be involved in the specification of progenitor cells in the human ganglionic eminences.

### Developmental trajectories in the ganglionic eminences

In rodents, the ganglionic eminences give rise to different neuronal populations that populate multiple structures of the adult telencephalon (30). The MGE gives rise to GABAergic projection neurons for the globus pallidus, GABAergic interneurons destined to the striatum and cerebral cortex, and cholinergic neurons that remain in the basal telencephalon (31–33). The LGE primarily produces striatal medium spiny neurons (MSNs) and olfactory bulb interneurons (31, 32). Finally, the CGE generates GABAergic projection neurons for the amygdala and other limbic system nuclei as well as a diversity of GABAergic interneurons that settle in the cerebral cortex (34).

We investigated whether cell diversification among neurons derived from the human ganglionic eminences follow similar developmental trajectories than those described in rodents. We first used a three-dimensional rendering of the distribution of human ganglionic eminence cells in UMAP to identify the relationships between progenitor cells and neurons (fig. S4A and Movie S1). We noticed that while the majority of MGE, LGE and CGE neurons are spatially related to progenitor cells, one group of MGE neurons (which we named MGE-2) was not connected to any group of progenitor cells. This suggested that the progenitor cells of MGE-2 neurons might not have been captured in our dataset, perhaps because they predate the first stage (GW9) we examined. We found that MGE-2 neurons express *NKX2-1, LHX8, GBX2*, and *ISL1* (fig. S4B), a combination of genes that is characteristic of subpallial projection neurons, which in the mouse are among the earliest neurons generated in the ganglionic eminences (35, 36). Consistent with this notion, we observed that MGE-2 neurons largely derive from the GW9 and GW12 samples (fig. S4C), and that cells with these features are already present in the human subpallium at GW8 (figs, S4, D and E).

**Fig. 3:**
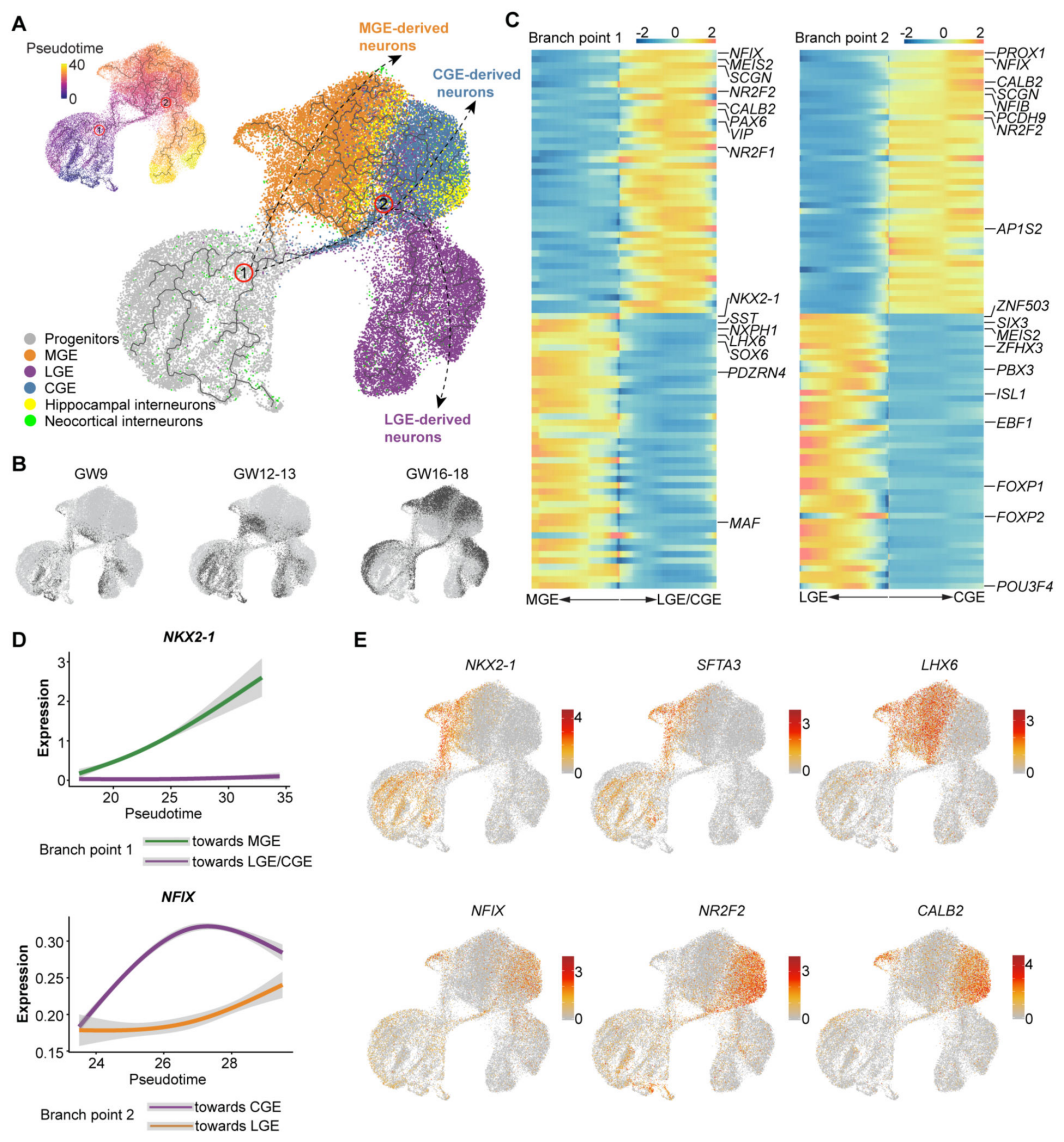
Transcriptional programs underlying the developmental divergence of human ganglionic eminence. (**A**) The cells of human ganglionic eminences and developing cortical and hippocampal interneurons are integrated and visualized by UMAP with inferred trajectories. MGE-2 cells were excluded from this analysis. Potential differentiation trajectories are schematically depicted with arrows. The pseudotime of ganglionic eminence cells is visualized via UMAP (top left). (**B**) Cell distributions at different gestational stages are shown in dark gray. (**C**) Heatmaps illustrating genes linked to cell fate divergence at branch point 1 (left) and 2 (right). (**D**) The expression of NKX2-1 and NFIX diverged at branch point 1 and 2, respectively. (**E**) The expression profile of genes related to cell fate divergence at branch point 1 and 2 is visualized by UMAP. Each dot represents an individual cell colored according to the expression level (red, high; grey, low).

We next integrated our dataset with published scRNA-seq datasets of human neocortical and hippocampal interneurons from GW8-27 (19, 20, 37), applied trajectory inference methods, and displayed the results via UMAP (Fig. 3, A and B). We excluded MGE-2 neurons from this analysis because their progenitor cells were not likely captured in our dataset. We found that most interneurons isolated from the developing neocortex and hippocampus cluster together with MGE and CGE neurons (Fig. 3A and fig. S5A), which is consistent with the view that most cortical interneurons derive from these structures (17). Referring to the inferred pseudotime trajectory, we identified the branch points that describe significant divergences in GE cells based on discrepant gene expression (Fig. 3A and fig. S5B). It revealed that the postmitotic cells in the ganglionic eminences first diverge into distinct MGE and LGE/CGE lineages at branch point 1, and that the later lineage subsequently segregates into separate LGE and CGE trajectories at branch point 2 (Fig. 3A).

We then investigated the transcriptional programs driving the divergent developmental trajectories of these cells at each of the branching events. We found pleiotropic genes potentially driving the divergence of cells along each developmental trajectory (Fig. 3C and + Table S7). For example, *NKX2-1, LHX6*, and *NXPH1* are enriched in cells following the MGE trajectory at branch point 1, while *PAX6, MEIS2*, and *NR2F2* are prevalent in cells within the LGE/CGE branch (Fig. 3, D and E and fig. S5, C and D). We also identified genes enriched in cells following LGE (*ZFHX3, FOXP1*, and *EBF1*) and CGE (*NFIX, PROX1*, and *NR2F2*) trajectories at branch point 2, respectively (Fig. 3, D and E and fig. S5, C and D).

### Transcriptional control of cell specification in the LGE

**Fig. 4:**
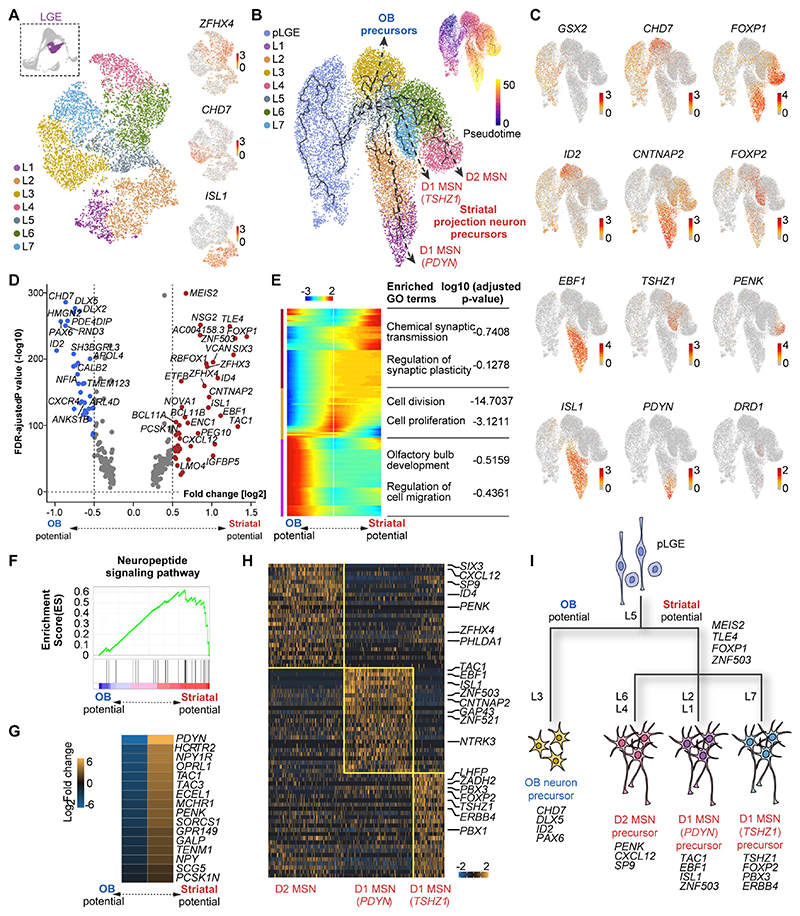
Transcriptional regulation of LGE development. **(A)** Unsupervised clustering of human LGE postmitotic cells and gene expression patterns visualized by t-SNE. Each dot represents an individual cell colored according to the expression level (red, high; grey, low). (**B**) Developmental trajectory of LGE cells visualized by UMAP. Pseudotime for individual cells is also shown (top right). (**C**) Gene expression profile along the developmental trajectories of LGE cells visualized via UMAP. (**D**) Volcano plot of DEGs for LGE cells with OB and striatal potential. (**E**) Heatmap illustrating the bifurcation of gene expression along the developmental trajectory of LGE cells committed to OB and striatal fates. (**F** and **G)** The neuropeptide signaling pathway is enriched in LGE cells with striatal potential. (**H**) Heatmap illustrating DEGs enriched in THSZ1+ D1 MSN, PDYN+ D1 MSNs and D2 MSNs. (**I**) Schematic of hypothetical genetic programs underlying the early diversification of human LGE cells.

We next sought to unveil the developmental trajectories of neurons generated in specific regions of the human ganglionic eminences. We first classified human postmitotic LGE cells using unsupervised clustering and performed differential gene expression analysis among the 7 resulting clusters (Fig. 4A and figs. S6, A and B). We found that clusters L1 and L2 contain cells expressing *ISL1, EBF1* and *TAC1*, which are enriched in striatonigral (D1) MSNs, whereas L4 primarily consisted of cells expressing *PENK*, a marker of striatopallidal (D2) MSNs. In addition, we observed that genes involved in the development of OB interneurons, such as *CHD7, ID2* and *PAX6*, were enriched among L3 cells (Fig. 4A and figs. S6, A and B).

We combined LGE progenitors and postmitotic cells to delineate their developmental trajectories and displayed the results via UMAP (Fig. 4B and fig. S6C). The results of this analysis revealed an early bifurcation of OB and striatal neuron fates which are delineated by characteristic patterns of gene expression (Fig. 4, B and C). We performed differential gene expression analysis to identify genes potentially driving the divergence of OB and striatal lineages (Fig. 4D and Table S8). Gene Ontology (GO) analysis on the DEGs revealed that GO terms associated with synaptic transmission and plasticity are enriched in LGE cells with striatal potential, while GO terms associated with OB development and cell migration are enriched in cells with OB potential (Fig. 4E).

We performed gene set enrichment analysis and found that the neuropeptide signaling pathway was distinctively enriched in the LGE cells with striatum potential (Fig. 4F and G). We also observed that striatal-specific genes such as *EBF1* and *ISL1* were expressed at earlier stages of development than OB-specific genes like *CHD7* (fig. S6D), which suggested that LGE cells with striatal potential mature earlier than cells with OB potential. To test this hypothesis, we defined a ‘maturation score’ and mapped the trajectory of LGE postmitotic cells along this inferred trajectory (fig. S6E). This analysis confirmed higher maturation levels for cells with striatum potential compared to cells with OB potential. Further analysis of the developmental trajectories of LGE cells with striatal potential revealed early divergence in three distinct fates, TSHZ1+ D1, PDYN+ D1 and D2 MSNs (Fig. 4B), each with a unique pattern of gene expression (Fig. 4H). Altogether, our analysis revealed the genetic programs underlying the early diversification of human LGE cells (Fig. 4I).

### Transcriptional control of cell specification in the MGE

**Fig. 5:**
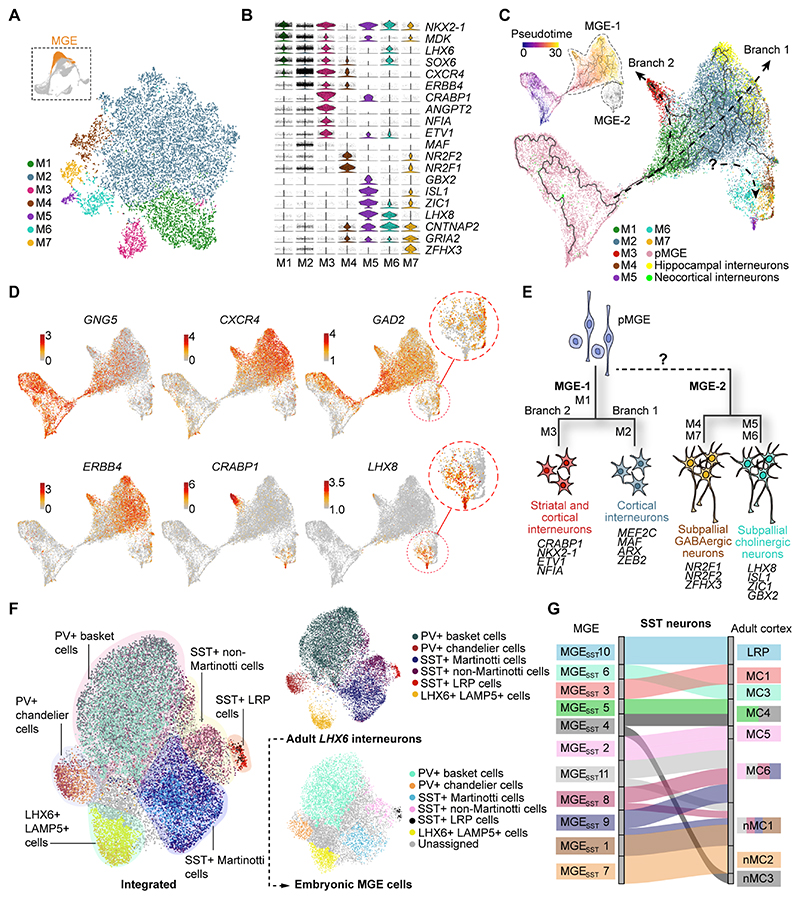
Transcriptional control of early cell specification in the human MGE. (**A**) Neuronal diversity of postmitotic cells in human MGE visualized via t-SNE. (**B)** Violin plots of DEGs among MGE clusters. (**C**) Developmental trajectory of MGE cells (including MGE progenitors and postmitotic cells) are inferred via monocle analysis and visualized by UMAP. Pseudotime of MGE cells in branch 1 and 2 is shown (top left). (**D**) Gene expression along the developmental trajectories of MGE cells is visualized via UMAP. MGE-2 cells comprise GABAergic and cholinergic subpallial neurons according to the expression of *GAD2* and *LHX8*, respectively. Each dot represents an individual cell and colored according to the expression level (red, high; grey, low). (**E**) Schematic of hypothetical genetic programs underlying the early diversification of human MGE cells. The developmental trajectory linking pMGE to MGE-2 cells is uncertain (dotted line) due to the lack of related progenitor cells in our dataset. **(F)** Integration of postmitotic human MGE cells (M2 and M3) and *LHX6*+ adult human cortical interneurons is visualized by UMAP. MGE cells were annotated according to the classification of adult cortical interneurons based on transcriptional similarities. (**G**) Riverplot illustrating the relationship between MGE cell clusters and adult SST+ interneurons. The size of the bars of MGE cells is normalized to cell numbers. LRP, long-range projection neurons; MC, Martinotti cells; nMC, non-Martinotti cells.

To explore neuronal diversity among newborn MGE cells, we classified postmitotic cells from this region using unsupervised clustering (Fig. 5A) and performed differential gene expression analysis among the 7 resulting clusters (Fig. 5B and figs. S7, A and B). We found distinctive patterns of gene expression among these cell populations. For example, the largest cluster M2 contains cells expressing *LHX6* and *SOX6*, two transcription factors that are critical for the development of MGE-derived cortical interneurons (38–41), and *CXCR4* and *ERBB4*, which encode guidance receptors regulating the tangential migration of interneurons from the ganglionic eminences towards the developing cortex (42–46). In contrast, M5 contains cells expressing *NKX2-1, LHX8, ISL1* and *GBX2*, which are characteristic of cholinergic neurons in the subpallium (Fig. 5B) (33).

To determine developmental relationships among these clusters, we integrated MGE progenitors and postmitotic neurons (with the exception of MGE-2 cells) with the datasets of developing human cortical and hippocampal interneurons, applied trajectory inference methods, and displayed the results via UMAP (Fig. 5C). This analysis revealed that M1 primarily contains undifferentiated precursors that are transitioning towards the acquisition of a distinct cell fate (Fig. 5C). The remaining clusters segregated along two separate trajectories. Branch 1 primarily contained cells from cluster M2 along with the majority of developing neocortical and hippocampal interneurons (Fig. 5, C and D). Branch 2 consisted of the small M3 cluster, which contains cells expressing *ETV1, CRABP1* and *ANGPT2* (Fig. 5, C and D, and fig. S7B). This cluster also exhibited high levels of *CXCR4* and *ERBB4* expression (Fig. 5D), which suggested it may include neurons tangentially migrating to the cortex and is consistent with the fact that some more mature neocortical and hippocampal interneurons also mapped to this branch (Fig. 5C). We also analyzed cell diversity within MGE-2 cells, which comprised a heterogeneous group of cells from the M4, M5, M6 and M7 clusters. We found very few neocortical and hippocampal interneurons among MGE-2 cells (Fig. 5C), which reinforced the idea that cells in these clusters are fated to develop into subpallial neurons. Consistently, we found that these cells segregated into prospective GABAergic (M4 and M7) and cholinergic (M5 and M6) fates with *GAD1* and *LHX8* expression, respectively (Fig. 5, D and E), which likely correspond to globus pallidus and cholinergic projection neurons of the basal telencephalon, along with a small number of striatal interneurons. Further analysis of differential gene expression revealed unique transcriptional programs that underlie the main developmental trajectories of diversification of human MGE neurons (Fig. 5E).

### Early specification of cortical interneurons

As many 60 different transcriptional identities have been identified among GABAergic neurons in the cerebral cortex of mice and humans (11, 12). To investigate the developmental mechanisms underlying the emergence of interneuron diversity in the adult human neocortex, we focused on the MGE, which gives rise to two main classes of cortical GABAergic neurons, parvalbumin-expressing (PV+) and somatostatin-expressing (SST+) cells (47). To this end, we integrated cells from the previously identified embryonic M2 and M3 clusters, which likely contain the majority of MGE-derived tangentially migrating interneuron precursors, with *LHX6*+ cells from two published single-nucleus RNA-seq datasets of the adult human cortex (11, 12) (fig. S8A), and visualized the combined dataset via UMAP (Fig. 5F and fig. S8B). Based on gene expression profiles, adult *LHX6*+ neurons were allocated to three main classes, *LHX6*+ *LAMP5*+, *PV*+, *SST*+ cells (fig. S8B), which could be further subdivided into multiple subclasses, including *PV*+ basket cells, *PV*+ chandelier cells, *SST*+ Martinotti cells, *SST*+ non-Martinotti cells, and *SST*+ long-range projection neurons (Fig. 5F). We then used this classification of adult neurons to annotate M2 and M3 MGE cells based on their transcriptional similarity and found that the majority of these cells could be assigned to one of the 6 subclasses identified in the adult cortex (Fig. 5F). Differential gene expression analysis among the embryonic neurons fated to become distinct subclasses of *PV*+ and *SST*+ neurons led to the identification of early developmental markers for these populations, many of which continued to be expressed in the corresponding adult interneuron subclasses (fig. S8C). This analysis suggested that interneuron diversification, at least at the level of the main interneuron subclasses, is evident in the human MGE long before these cells reach the developing cortex.

We next searched for evidence of fate specification at the level of individual cell types among human MGE cells. To this end, we focused on *SST*+ neurons as they are perhaps the best characterized among the different transcriptional identities observed in the adult cortex (11, 12). We used unsupervised clustering to classify adult SST+ neurons into 13 unique transcriptional identities: one type of long-range projection neuron, six types of Martinotti cells, and six types of non-Martinotti cells (fig. S9A-F). We then integrated the adult and embryonic datasets and assigned prospective cell-type identities to embryonic *SST*+ cells based on their transcriptional similarity with adult SST+ cell-types (fig. S9G). Using this approach, we identified 11 distinct transcriptional identities (MGE_SST_ 1-11) among the embryonic SST+ interneurons which exhibit unique patterns of gene expression (fig. S9, G and H). We also conducted an independent, pairwise comparison analysis to define homologies among the embryonic and adult SST+ identities. This analysis revealed that 7 of the 11 embryonic SST+ transcriptional identities identified in the MGE (MGE_SST_ 1, 2, 3, 5, 6, 7, and 10) matched one-to-one with adult SST+ transcriptional cell-types, including one type of long-range projection neuron, four Martinotti cell-types, and two non-Martinotti cell-types (Fig. 5G). Our analysis also revealed that four of the embryonic SST+ transcriptional identities (MGE_SST_ 4, 8, 9 and 11) have similarities with both the Martinotti cell and non-Martinotti cell lineages (Fig. 5G), perhaps reflecting a relatively immature state. Furthermore, we could not identify embryonic correlates of four adult SST+ transcriptional identities, which are primarily defined by the expression of ion channel genes barely detectable in embryonic interneurons (fig. S9, E and F). Altogether, these results suggest that individual interneuron cell types –as defined by their transcriptional identity– are specified shortly after birth in the human embryonic MGE.

### Unique features in developing human interneurons

**Fig. 6:**
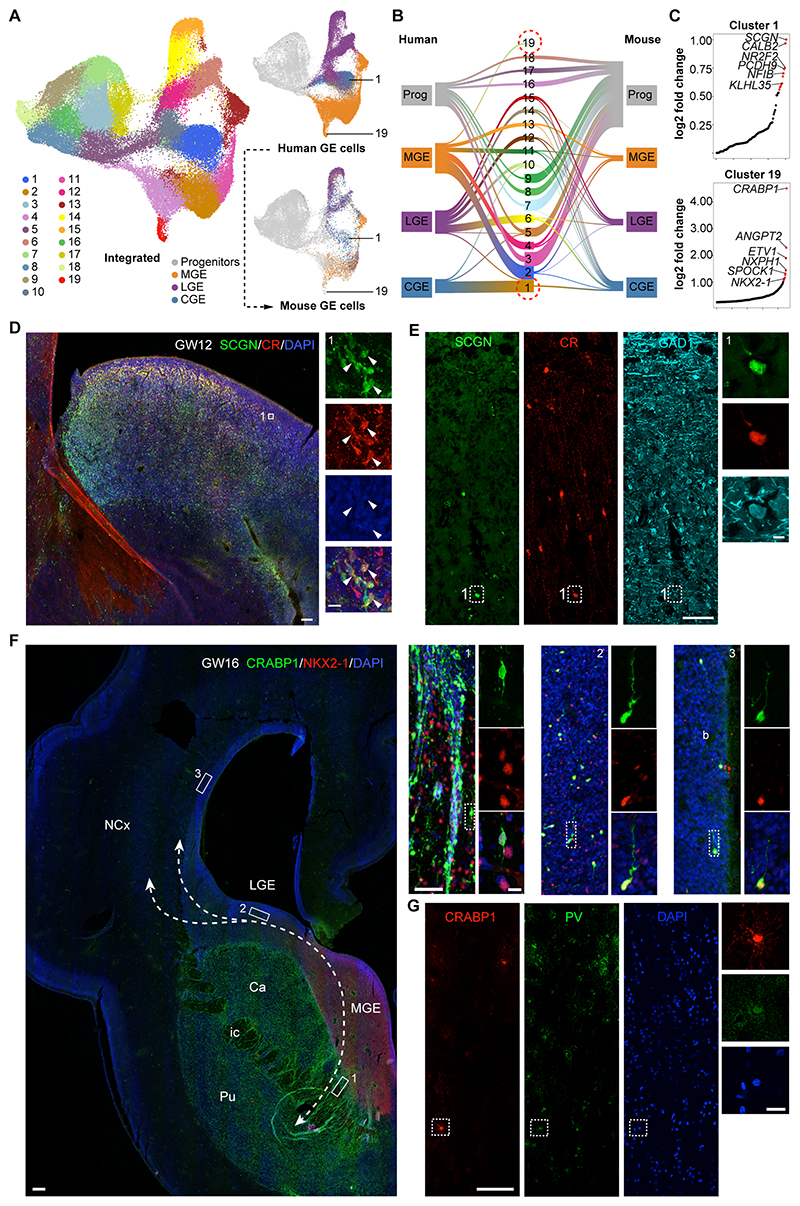
Unique features of human ganglionic eminences. (**A**) The datasets of human and mouse ganglionic eminence cells are integrated based on shared sources of variation and visualized via UMAP. (**B**) Riverplot illustrating relationships between human and mouse ganglionic eminence cell clusters. Two human-specific clusters (1 and 19) are highlighted by circles. (**C**) Specific gene expression in human-specific clusters 1 and 19. (**D**) Expression of SCGN and CR in the human CGE at GW12. The area in the white box is shown at high magnification. Scale bars, 100 µm (left), 20 µm (right). (**E**) Expression of SCGN, CR and GAD1 in the adult human cortex. The area in the white box is shown at high magnification. Scale bar, 100 µm (left), 10 µm (right). (**F**) Expression of CRABP1 and NKX2-1 in the embryonic human brain at GW16. The regions in the white boxes are shown at high magnification. The dotted lines illustrate potential migratory routes for CRABP1+ and NKX2-1+ cells. Scale bar, 500 µm (left), 50 µm (boxes 1-3, right), 10 µm (boxes 1-3, left). (**G**) Expression of CRABP1 and PVALB in the adult human cortex. The area in the white box is shown at high magnification.

Despite sharing many cellular features with other species, human cortical interneurons also exhibit some distinctive traits, including morphological and gene expression specializations (11, 12). To investigate whether any of these unique characteristics are already embedded in the ganglionic eminences during embryonic development, we integrated our scRNA-seq dataset of human ganglionic eminence cells (GW9-18) with two published datasets of mouse ganglionic eminence cells from embryonic days 12.5-14.5 (48, 49) (figs. S10, A and B) and identified 19 cell groups using unsupervised clustering (Fig. 6A, fig. S10C and Table S9). We then calculated the normalized cell ratio of human and mouse cells for each cluster and detected two clusters, 1 and 19, which primarily consisted of human CGE and MGE cells, respectively (Fig. 6B and fig. S10D). We analyzed gene expression in these two predominantly human cell groups and found that cells in each of these clusters exhibit specific markers, such as *SCGN, CALB2* and *NR2F2* in cluster 1, and *CRABP1, ETV1* and *NKX2-1* in cluster 19 (Fig. 6C).

We classified human postmitotic CGE cells into three main groups (C1-3) using unsupervised clustering (fig. S10E). Cell constitution analysis revealed that the majority of CGE cells at these stages belong to C1 and C2, which exhibit the highest levels of *SCGN* and *CALB2* expression and correspond to cluster 1 (fig. S10F). Immunohistochemical analyses confirmed that cells expressing secretagogin and calretinin, the proteins encoded by *SCGN* and *CALB2*, respectively, concentrate in the SVZ of the human CGE, where they often colocalize with NR2F2 (Fig. 6D and fig. S11A). In contrast, we detected very few SCGN+ cells in the mouse CGE (fig. S11B). In the adult human cortex, secretagogin-expressing neurons are GABAergic and often contain calretinin (Fig. 6E and fig. S11C).

We performed cell constitution analysis for cluster 19 and found that it mainly consisted of M3 cells, characterized by the expression of *CRABP1* and *NKX2-1* (fig. S10G). At GW16, cells co-expressing CRABP1 and NKX2-1, with the typical morphology of migrating interneurons (46, 50), were found in the striatum and en route towards the pallium (Fig. 6F). In addition, CRABP1/NKX2-1+ cells were also found in the developing human neocortex (Fig. 6F and fig. S12A). Crabp1/Nkx2-1+ cells could be detected in the mouse subpallium, but not in the developing or adult cortex (figs. S12, B and C). In contrast, CRABP1+ cells co-expressing PV were detected in the adult human cortex (Fig. 6G), which suggests they may represent a population of fast-spiking interneurons.

## Discussion

In this study, we investigated the transcriptional identity of cells in the developing human ganglionic eminences as well as the gene regulatory networks controlling their fate specification. Using single-cell transcriptomics and trajectory inference methods, we built spatiotemporal maps of gene expression through the early second trimester of human development (GW9-18) that recapitulate the developmental trajectories of the main classes of neurons generated in the subpallium, including olfactory bulb neurons, striatal and pallidal GABAergic projection neurons, cholinergic projection neurons and interneurons, and striatal and cortical GABAergic interneurons. Our results revealed conserved mechanisms underlying the early diversification of subpallial neurons in mouse and human. Although the specific expression of some key factors such as *NKX2-1, SIX3, SCGN, CALB2, CRABP1, ISL1* and *LHX8* in the developing human brain has been validated via immunostaining, further experiments should investigate their precise involvement in this process.

Newborn neurons are generated from RGCs and IPCs in both ganglionic eminences and developing cortex. The larger primate neocortex is due to a greater number of RGCs, especially basal RGCs in the SVZ, which in rodents is mostly populated by IPCs (14, 51, 52). Although it has been suggested that a shift towards more basal RGCs may also drive the greater neurogenesis in human than in mouse ganglionic eminences (16), we found that the massive growth of the human ganglionic eminence SVZ during the second trimester is primarily due to greater numbers of IPCs. Thus, for the human, the main progenitor cell populations driving neurogenesis in the ganglionic eminences are different than in the developing cortex.

Previous work has shown that the expression patterns of several transcription factors in the primate ganglionic eminences are very similar to those found in rodents (17, 29). Our transcriptomic analysis reveals that these conserved features extend beyond a handful of key factors and include the complex gene regulatory networks controlling neuronal specification in the human ganglionic eminences. Cortical interneurons in the human ganglionic eminences express the same receptors that have been shown to regulate the tangential migration of interneurons in rodents (53). This reveals that the conserved features of human and mouse interneuron development extend beyond the early specification of progenitor cells and involve essential aspects of their subsequent migration and differentiation. Although the roles of some key transcription factors in orchestrating interneuron development have been studied in the mouse, their function in human interneuron development requires experimental validation.

Cortical interneurons are a heterogeneous group of neurons with diverse morphologies, connectivity, biochemistry, and physiological properties (54, 55). In mice, newborn interneurons are transcriptionally heterogeneous within a few hours of becoming postmitotic, and these early transcriptional signatures correlate with those found in specific types of interneurons in the adult cortex (49) Our study reveals that interneuron diversification also begins in the human ganglionic eminences, long before these cells reach the developing cortex. For instance, many of the transcriptional identities found among SST+ interneurons in the adult human cortex are identifiable in the MGE. In some cases, however, the transcriptional signature of developing interneurons does not allow a clear correlation with specific adult identities. This is the case of newborn CGE interneurons, most of which are jointly characterized by the expression of *CALB2* and *SCGN*, much like developing CGE-interneurons in rodents are characterized by the expression of *Htr3a* (56). *SCGN* encodes a calcium-binding protein similar in sequence and structure to calbindin and calretinin which seems particularly abundant in the developing human brain (57, 58). In the adult human cortex, *SCGN* expression seems to be confined to a small population of GABAergic interneurons whose function remains to be established. We also identified a cluster of human MGE-derived interneurons characterized by the expression of *CRABP1* which does not seem to have a clear counterpart in rodents. In the mouse, *Crabp1* is expressed by some fast-spiking interneurons in the developing striatum (59). In the human, we found migrating CRABP1-expressing interneurons end up in the adult human cortex and co-express NKX2-1 and PV. This observation suggests that CRABP1+ cells may represent a population of MGE-derived fast-spiking interneurons in the human cortex.

Although the general cellular architecture of inhibitory cell types is largely conserved, extensive differences seem to exist in the relative proportions, laminar distributions, and gene expression patterns of specific types of interneurons in the adult cortex of mice and humans. For instance, *LHX6*+ *LAMP5*+ interneurons are much more abundant in the human than in the mouse adult cortex (11, 12). Our findings reveal that human *LHX6*+ *LAMP5*+ interneurons originate in the MGE like their mouse counterparts and shared a common developmental trajectory, which suggests that the differences observed between mouse and human may arise through changes in the dynamics of neurogenesis (16) or in the interaction of developing interneurons with the local microenvironment, for instance via programmed cell death (60).

Neurodevelopmental disorders have overlapping phenotypes and genetics, suggestive of common deficits. Changes in striatal and cortical GABAergic neurons have been extensively documented in autism and schizophrenia (61), yet it is presently unclear whether genetic variation associated with these disorders converges on specific types of inhibitory cells because we lack a detailed understanding of their transcriptional trajectories in the developing human brain. Our study sheds light on the development of human GABAergic neurons and should enable linking gene variation and cell types in neurodevelopmental disorders.

## Materials and Methods Summary

Detailed materials and methods are provided in the supplementary materials. In brief, human fetal ganglionic eminence (GE) samples across gestational weeks (GW) 9-18 were collected from Beijing Anzhen Hospital with approval from the Reproductive Study Ethics Committee of Beijing Anzhen Hospital and the institutional review board (ethics committee) of the Institute of Biophysics. Single-cell RNA-sequencing was performed with an Illumina sequencing platform. The outcome reads were aligned to the human reference genome hg19 with Cell Ranger (10X genomics). Doublets were removed via performing the scrublet pipeline on the raw gene-by-cell expression matrix from each sample (62). Batch correction was conducted on the reduced dimensions with the R function of fastMNN (63). Seurat was adopted to perform normalization, dimension reduction, unsupervised clustering and differentially expressed genes identification (64, 65). The Seurat integration method was used to determine the developmental divergence of human GE cells, the early specification of cortical interneurons in human GE, as well as unique features of developing human interneurons. The developmental trajectory of human GE cells was constructed via the R package of monocle 3 (66–68). K-nearest neighbors analysis (knn) was used to render the putative interneuron identities to MGE cells. Immunofluorescence staining was performed on human and mouse brain slices and images were acquired with an Olympus confocal microscope.

## Supplementary Material

Supplementary Materials

## Data Availability

The single-cell RNA-seq data used in this study have been deposited in the Gene Expression Omnibus (GEO) under the accession number GSE135827. The Supplementary Materials contain additional data. All data needed to evaluate the conclusions in the paper are present in the paper or the Supplementary Materials.
