# Supplementary material for: Mouse and human share conserved transcriptional programs for interneuron development
Source: Science. Author manuscript; Available in PMC 2025 Oct 11. (PMC7618238; doi:10.1126/science.abj6641)
Supplement: Supplementary Materials [file EMS209515-suppement-Supplementary_Materials.pdf]

SUPPLEMENTARY MATERIALS

Materials and Methods

Figs.S1 to S12

Tables S1 to S9

References (69-75).

Supplementary Materials for

Mouse and human share conserved transcriptional programs for interneuron development

Yingchao Shi[1,*], Mengdi Wang[1,2,*], Da Mi[3,4,5,*], Tian Lu[1,2], Bosong Wang[6], Hao Dong[1,2], Suijuan Zhong[6,7], Youqiao Chen[6], Le Sun[8], Xin Zhou[1], Qiang Ma[1,2], Zeyuan Liu[1,2], Wei Wang[1,2], Junjing Zhang[6], Qian Wu[6,7,†], Oscar Marín[4,5,†], Xiaoqun Wang[1,2,†]

*These authors contributed equally to this work
†Corresponding authors. Email: xiaoqunwang@ibp.ac.cn (XW); oscar.marin@kcl.ac.uk (OM); qianwu@bnu.edu.cn (QW)

This PDF file includes:

Materials and Methods
Figs. S1 to S12
Tables S1 to S9
References 69 to 75

Materials and Methods

Human subjects

The human tissue collection and research protocols were approved by the Reproductive Study Ethics Committee of Beijing Anzhen Hospital and the institutional review board (ethics committee) of the Institute of Biophysics. The fetal tissue samples were collected after the donor patients signed an informed consent document which is in strict observance of the legal and institutional ethical regulations of Beijing Anzhen Hospital.

Animals

CD1 mice at embryonic day 13.5 and postnatal day 60 were used for immunostaining studies. The animals used for experiments include both male and female mice. Mouse housing and experimental protocols in this study were in compliance with the guidelines of the Institutional Animal Care and Use Committee of the Institute of Biophysics, CAS. All mice had free access to food and water and were housed in the institutional animal care facility with a 12-h light–dark schedule.

Tissue Dissection, Single-Cell Dissociation and Single-cell RNA-seq library preparation

Human fetal ganglionic eminence samples across GW9-GW18 were collected in ice-cold artificial cerebrospinal fluid (ACSF). For the sample of GW18-02, the medial, lateral and caudal ganglionic eminences (MGE, LGE and CGE, respectively) were regionally dissected under a dissection microscope. The human fetal tissue was subsequently dissociated into single-cell suspension using a papain-based dissociation protocol (hibernate E medium with 1 mg/ml papain (Sigma) at 37°C on a thermocycler with 500g for 15-20 min). Single cells were suspended in 0.04% BSA/PBS at the proper concentration to generate cDNA libraries with Single Cell 3' Reagent Kits, according to the manufacturer's protocol. Briefly, after the cDNA amplification, enzymatic fragmentation and size selection were performed to optimize the cDNA size. P5, P7, an index sample, and R2 (read 2 primer sequence) were added to each selected cDNA during end repair and adaptor ligation. P5 and P7primers were used in Illumina bridge amplification of the cDNA (http://10x genomices.com). Finally, the library was processed on the Illumina platform for sequencing with 150 bp pair-end reads.

Single-cell RNA-seq data processing

Barcode filtering and sequence alignment were performed with software Cell Ranger (http://10xgenomics.com) (*69*). Reads were aligned to the human reference genome (hg19). To detect potential doublets, the scrublet (v0.2.1) (*62*) pipeline was performed on each sample by setting parameters 'expected_doublet = 0.06', 'sim_doublet_rate=20, min_gene_variability_pctl=85' and 'n_prin_comps=30'. 231/61488 cells with computed doublet score greater than the doublet score threshold were identified as doublets and excluded from subsequent analysis. Next, the filtered cell-by-gene count matrix was loaded into Seurat (*63, 64*) for downstream analysis. We excluded cells that did not meet the following criteria: 1) cells with number of detected genes greater than 800; 2) cells with number of expressed genes between 800 and 4000; 3) cells with UMI (unique molecular identifier) smaller than 15000; 4)

cells with percentage of mitochondrial counts smaller than 5; 5) cells with percentage of hemoglobin counts smaller than 5. Genes that express in less than 5 cells were removed. Overall, 22784 genes and 56412 cells were retained for subsequent analysis. Next, we loaded the filtered count matrix to function of CreateSeuratObject to create a Seurat object followed by log-normalization of the count matrix by NormalizeData function. The top 2000 variable genes were identified using the FindVariableFeatures function. Then, principal component (PCA) analysis was performed using the RunPCA function. Batch effect correction was conducted on the principal components with function fastMNN (*63*). Unsupervised clustering was performed with the function of FindNeighbors and FindClusters. Uniform manifold approximation and projection (UMAP) was employed for visualization of clustering with the RunUMAP function.

Identification of differentially expressed genes among clusters

Differential gene expression analysis among clusters was performed using the Seurat FindAllMarkers function. Genes with adjusted P-values <0.05 were selected as differentially expressed genes (DEGs).

Construction of single-cell developmental trajectory

For the construction of single-cell developmental trajectories and discovering trajectory transitions, the R package monocle3 (*66-68*) was applied. We converted the Seurat object into a SingleCellExperiment object and ordered cells along pseudotime trajectory with the function of order_cells.

Plotting the landscape of gene expression in pseudo-lamina and pseudo-differentiation space

To compute pseudo-lamina score, we firstly identified lamina related genes by correlating variable genes with a binarized vector representing lamina variables, such that the score of 1 and 2 labels cells from VZ and SVZ, respectively. Variable genes with a correlation coefficient greater than 0.2 were then used for a subsequent linear regression analysis. Next, we performed a principal component analysis with genes that highly correlated with lamina variables (e.g., correlation coefficient greater than 0.2) using the RunPCA function. To compute the pseudo-differentiation score, we constructed a single-cell developmental trajectory and ordered cells in pseudotime by performing the monocle analysis pipeline. To infer gene expression profile in the space of pseudo-lamina and pseudo-differentiation, we constructed a surface plot along these two axes (pseudo-lamina and pseudo-differentiation) using the fit() function in Matlab with “lowess” smoothing model.

Dendrogram construction based on gene expression

To build a dendrogram of cell types, we performed a principal component analysis with the cell-by-gene expression matrix using function RunPCA and applied the BuildClusterTree function of Seurat on the resulted PCs. The PlotClusterTree function was employed for dendrogram visualization.

Developmental trajectories inferencing in the human ganglionic eminences

To analyze the potential developmental trajectories of human ganglionic eminence at the transcriptomic level, we integrated our human embryonic dataset (excluding MGE-2 cells) with the published scRNA-seq datasets of embryonic human neocortical and hippocampal interneurons (*19, 20, 37*). In brief, we first merged the cell-by-gene expression matrix from the three published datasets, which was then loaded to the Seurat pipeline for subsequent dimension reduction, clustering and cell-type annotation. Then, we identified variable features for the human embryonic dataset and the assembled neocortical and hippocampal interneuron datasets independently. Next, the embryonic dataset was integrated with the dataset of neocortical and hippocampal interneurons. The FindIntegrationAnchors and IntegrateData functions of Seurat were performed, followed by dimension reduction analysis using the RunPCA and RunUMAP functions of Seurat. Then, trajectory inference was performed to indicate the potential developmental trajectories of embryonic human cells based on gene expression, we ordered cells along the trajectory by performing monocle analysis. Monocle identifies branch points that describe significant divergences in the cellular state automatically. Thus, the pseudotime trajectory can be used to infer major differentiation paths based on the ramification in gene expression. Given the progenitor cells of MGE-2 cells are not captured in our dataset, we performed pseudotime trajectory inference on the ganglionic eminence cells apart from MGE-2 cells via monocle analysis.

Gene expression analysis along developmental trajectories

To identify key genes changes along the developmental trajectory of human ganglionic eminence cells, we first performed monocle analysis to order cells along pseudotime trajectory and then modeled gene expression as a smooth function with pseudotime value by applying a vector generalized additive model (VGAM) with R package VGAM as described previously (*70, 71*). Heatmap was applied for visualization of gene expression along a developmental trajectory.

Developmental trajectory inference in the human MGE cells

To infer the potential developmental trajectory of the human MGE cells, we integrated the MGE progenitor cells and postmitotic neurons with the developing neocortical and hippocampal interneurons that mapped to the MGE in Fig. 3A utilizing the Seurat functions of FindIntegrationAnchors and IntegrateData. Then, dimensionality reduction analysis was performed on the assembled dataset with the function of RunPCA and RunUMAP. Next, trajectory inference analysis was conducted on the integrated dataset with the monocle 3 pipeline. MGE-2 cells were not included in the trajectory inference and pseudotime analysis.

Construction of a maturation trajectory

To construct a maturation trajectory as described previously (*48*), we first performed dimensionality reduction with diffusion maps and fitted the resulted data onto a principal curve with the R Package of princurve. The length from the beginning of the curve to the point where the cell projects onto the curve was defined as the maturation score of each cell. To assign a direction for the curve, we correlated the gene expression of *GSX2* with the maturation score.

Cells with a negative correlation coefficient were ordered at the beginning of the maturation curve.

Mapping human embryonic MGE post-mitotic cells to adult cortical interneurons

To infer lineage identities of human embryonic ganglionic eminence cells, we mapped human embryonic MGE post-mitotic cells (cells of M2 and M3) to publicly available datasets of adult human cortical GABAergic interneurons from the studies of Allen Brain Institute (https://portal.brain-map.org/atlases-and-data/rnaseq) and Krienen and colleagues (*11*, *12*). Briefly, we first assembled the cell-by-gene expression matrix from the two datasets, which was then loaded into the Seurat pipeline for dimension reduction, clustering, and cell-type annotation. Next, we identified variable genes in both embryonic and adult datasets and conducted canonical correlation analysis (CCA) using computed variable genes with the Seurat functions of RunCCA and AlignSubspace. We then performed UMAP analysis for dimensionality reduction. Subsequently, k-nearest neighbors analysis (knn) was conducted with two UMAP coordinates (UMAP1 and UMAP2) using R function of knn by setting parameters k = 10 and l = 9. For each MGE post-mitotic cell, we counted the identities of its 10 closest cortical interneurons. If at least (k-l) (in our case, 9) interneurons have the same major cell type identities, the MGE cell would be assigned to that cell identity. Subsequently, we extracted the cells with assigned identities of SST or PV interneurons for downstream analysis and performed again the CCA and knn routines with adult subtype features (i.e., SST and PV interneuron subtypes) to assign embryonic cells a putative cortical GABAergic interneuron subtype identity. The interneuron identity of assigned cells was acquired and the contribution ratio was computed and visualized with alluvial plots.

Integration of human and mouse datasets

To compare the transcriptomic profiles of human and mouse embryonic ganglionic eminence cells, we assembled human (our scRNA-seq datasets) and mouse datasets from previously published studies (*48, 49*) using the AlignSubspace function of Seurat with shared variable genes in both the human and mouse datasets among the 1:1 orthologue. Cell clustering and dimensionality reduction were performed with FindClusters and RunUMAP function, respectively. To evaluate cell occupancy in each cluster, we computed the cell ratio of human to mouse ganglionic eminence cells in each cluster after normalizing the total cell numbers in each species. Groups with cell occupancy >2 or <-2 (that is, log2[cell ratio] > 2 or log2[cell ratio] < -2) were considered as human-predominant or mouse-predominant groups, respectively. A river plot was constructed to illustrate the mapping pattern of cells from human and mouse datasets.

Gene Ontology (GO) enrichment analysis

Gene Ontology (GO) enrichment analysis was performed with DAVID 6.7 (https://david.ncifcrf.gov) (*72, 73*) and Gene Set Enrichment Analysis (GSEA) (*74, 75*), respectively.

Immunofluorescence

Human fetal brains were fixed by 4% paraformaldehyde in PBS at 4°C and then dehydrated in 30% sucrose in PBS. The fixed and dehydrated tissues were embedded and frozen at -80 °C in O.C.T. compound. The human brain slices were sectioned with Leica CM3050S. Cryosections were subjected to antigen retrieval, pretreated (0.3% Triton X-100 in PBS) and incubated for a blocking solution (10% normal donkey serum, 0.1% Triton X-100, and 0.2% gelatin in PBS), followed by incubation with the primary antibodies overnight at 4°C. The following primary antibodies were used: CR (Swant, 6B3,1:200), CRABP1 (NOVUS,NB300-539,1:500), DCX (Abcam, ab18723, 1:300), FAM107A (Sigma, HPA055888,1:200)GAD1 (Abcam, ab75712, 1:200), Ki67 (Millipore, AB9260, 1:200), NKX2-1 (Abcam, ab86023, 1:200), NR2F2 (NOVUS,NB300-539,1:200), NTRK2 (R&D,AF1494,1:100), PAX6 (BioLegend, 901301, 1:200), PV (Millipore, AB15736, 1:200), SCGN (Abcam, ab111871,1:200), SIX3 (Abcam, ab221750,1:200), and VIM (Millipore CBL202, 1:200). Sections were then washed three times for 10 minutes with 0.1% PBST, before being incubated in the following secondary antibodies donkey anti-mouse 488 (1:500), donkey anti-rabbit 594 (1:500), donkey anti-rabbit 488 (1:500), donkey anti-goat 488 (1:500), donkey anti-chicken (1:500) diluted in blocking solution. Sections were finally counterstained with DAPI, before being mounted in series with Mounting medium (Sigma Aldrich). Immunofluorescence images were acquired with Olympus laser confocal microscope and analyzed with FV10-ASW viewer (Olympus), ImageJ (NIH) and Photoshop (Adobe).

Supplementary Figures

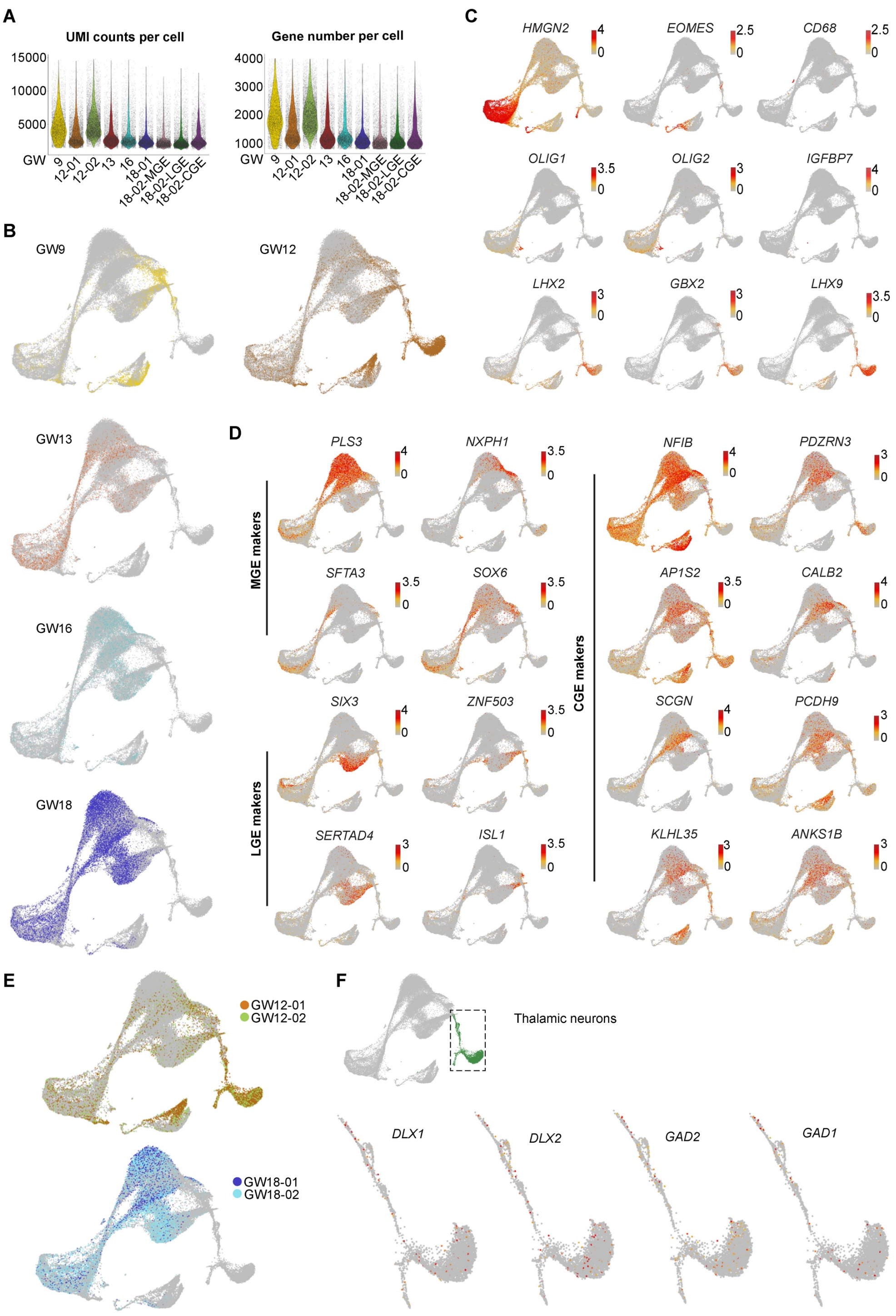

Fig. S1. Transcriptional features of human ganglionic eminence cells. (**A**) Quality control for the scRNA-seq dataset of human ganglionic eminence samples, each dot represents a single cell. Cells with gene number per cell (nGene) < 800 and > 4000 (upper), as well as

mitochondrial gene percentage > 5% (lower) were discarded in the following analysis. (**B**) The distribution of cells from different gestational weeks in UMAP is visualized individually. (**C**) The gene expression profile of well-known marker genes is visualized via UMAP. HMGN2, a marker of progenitor cells; EOMES, a marker of IPCs of excitatory neurons; CD68, a marker of microglia; OLIG1 and OLIG2, markers of OPC; IGFBP7, a marker of endothelial cells. LHX2, LHX9 and GBX2 are markers of thalamic neurons. Cells are colored according to gene expression levels (red, high; grey, low). (**D**) The expression pattern of gene markers of postmitotic cells in human MGE, LGE and CGE are visualized via UMAP. Each dot represents one cell and is colored according to gene expression level (red, high; grey, low). (**E**) The cell distribution of human ganglionic eminence samples collected at GW12 (GW12_01 and GW12_02) and GW18 (GW18_01 and GW18_02) visualized via UMAP. (**F**) DLX1/2 positive GABAergic neurons could be detected in the thalamic neurons.

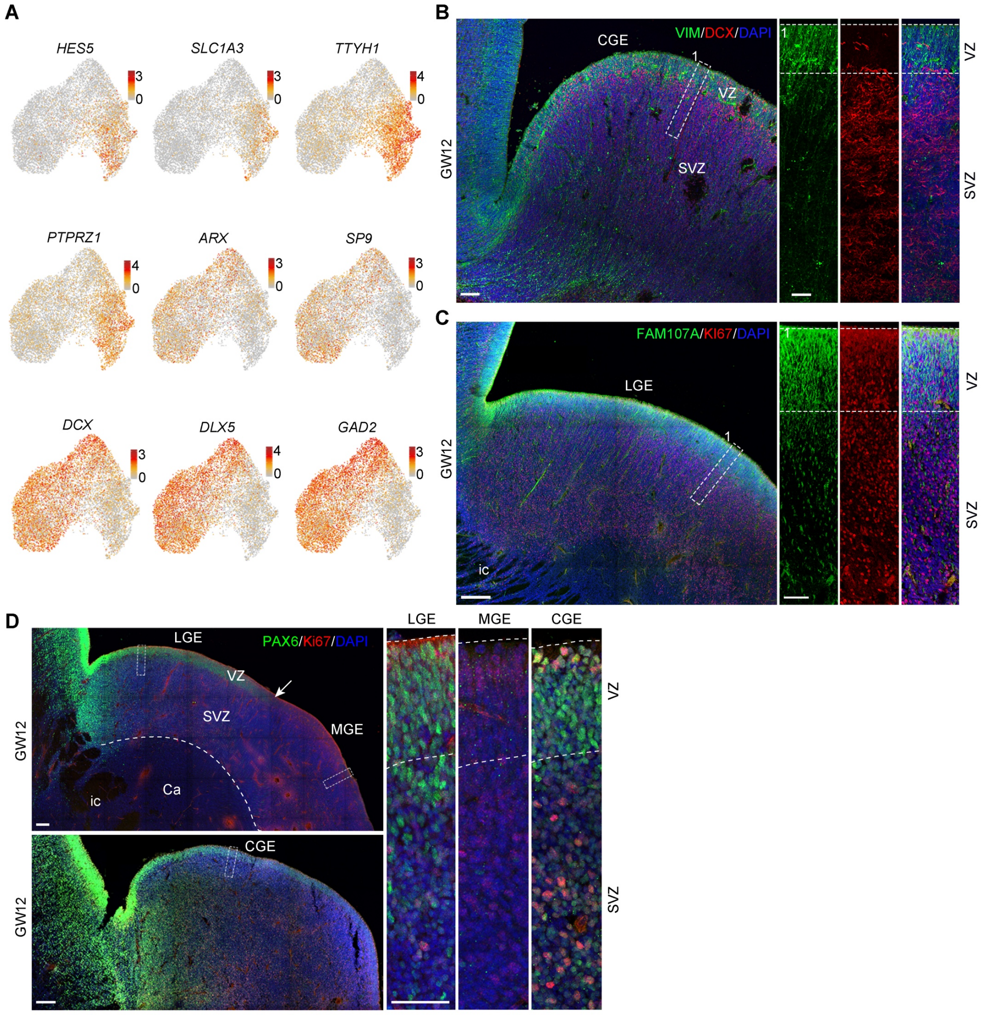

Fig. S2. Transcriptional profiles and spatial organization of progenitor cells in the human ganglionic eminences. (**A**) The expression profile of genes enriched in radial glia (RG) and intermediate progenitor cells (IPCs) in the human ganglionic eminences visualized via UMAP. Each dot represents one cell and is colored according to gene expression level (red, high; grey, low). (**B**) Immunofluorescence staining for VIM and DCX in the human ganglionic eminences at GW12. The expression of VIM is mainly restricted to the VZ, while DCX is mainly expressed in the SVZ. Scale bars, 100 µm (left), 50 µm (right). **(C)** Immunofluorescence staining for FAM107A and Ki67 at GW12. The expression of FAM107A is mainly restricted to the VZ. Scale bars, 100 µm (left), 50 µm (right). **(D)** Immunofluorescence staining for *PAX6* and *Ki67* in the MGE, LGE and CGE at GW12. Scale bars, 200 µm (left), 100 µm (right).

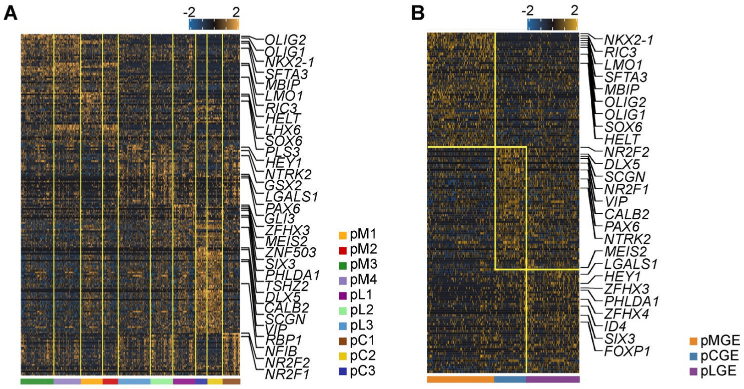

Fig. S3. Differential gene expression underlie the regional specification of progenitor cells in the human ganglionic eminences. (**A-B**) Heatmap illustrating the DEGs expression among subclusters of human ganglionic eminence progenitor cells (A) as well as among regionally-segregated progenitors (B).

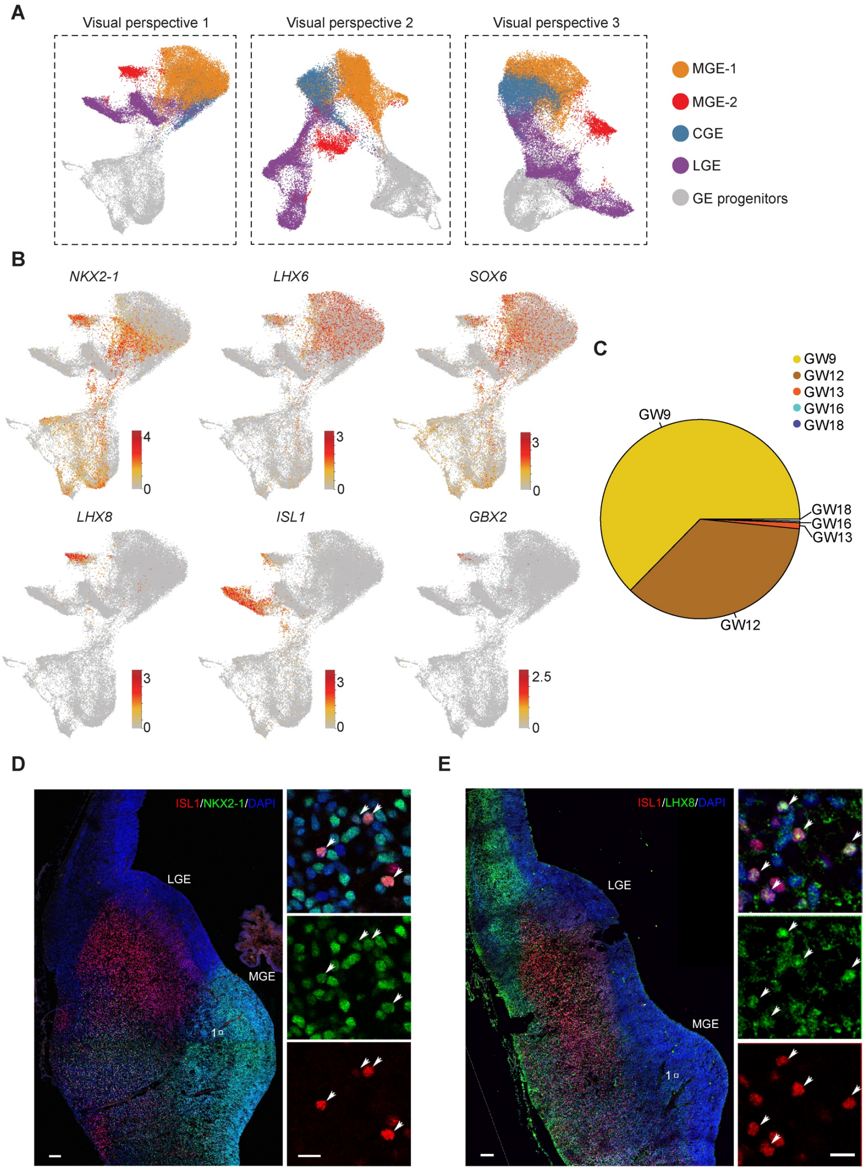

Fig. S4. MGE cells potentially fated to subpallial neurons are detected in the human ganglionic eminence dataset. (**A**) When visualized in 3D UMAP, a group of MGE cells (MGE-2 cells) is spatially segregated from other MGE cells (MGE-1). (**B**) The expression profile of genes characteristic of MGE cells (*NKX2-1*, *LHX6* and *SOX6*) and subpallial neurons (*LHX8*, *ISL1* and *GBX2*) in MGE-2 cells is visualized via UMAP. Cells are colored according to gene expression levels (red, high; grey, low). (**C**) Composition of MGE-2 cells from different gestational weeks. (**D-E**) Immunostaining for NKX2-1/ISL1 (D) and LHX8/ISL1 (E) in the human telencephalon at GW8. The areas in boxes are amplified in the right panels. Scale bar, 100 µm (left), 10 µm (right).

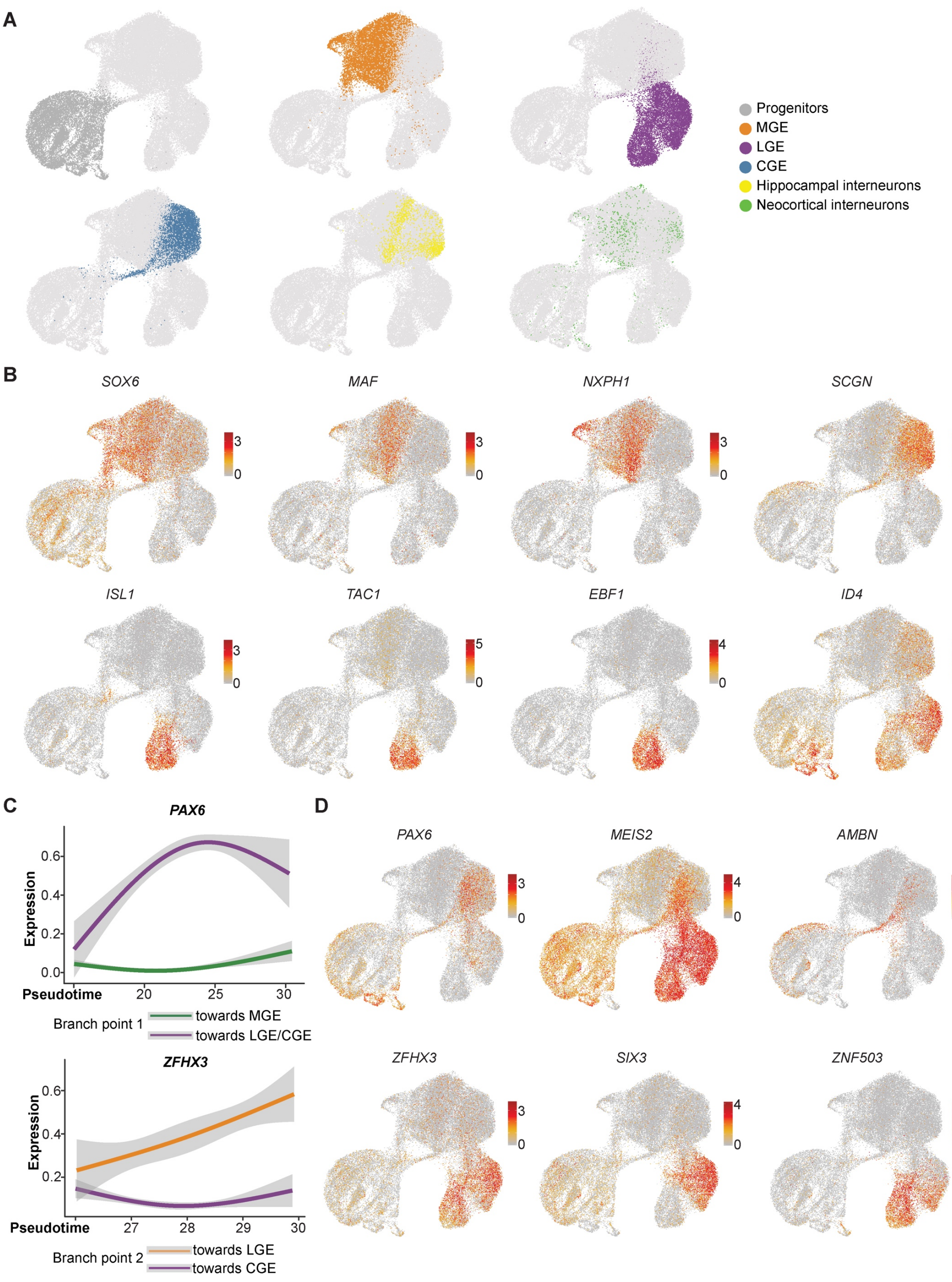

Fig. S5. Genetic mechanisms regulating developmental divergence in the human ganglionic eminences. (**A**) The distribution of different cell groups in the integrated dataset of ganglionic eminence cells and neocortical/hippocampal interneurons is shown individually in UMAP. (**B**) Expression profile of characteristic regional-identity genes in ganglionic eminence cells is visualized via UMAP. Cells are colored according to gene expression levels (red, high; grey, low). (**C**) The expression pattern of *PAX6* and *ZFHX3* in cells diverged at branch point 1 and 2 is shown via fitted curves along pseudotime. (**D**) The expression pattern of genes that may potentially play a role in regional specification of ganglionic eminence cells is visualized. Each dot represents an individual cell colored according to the expression level (red, high; grey, low).

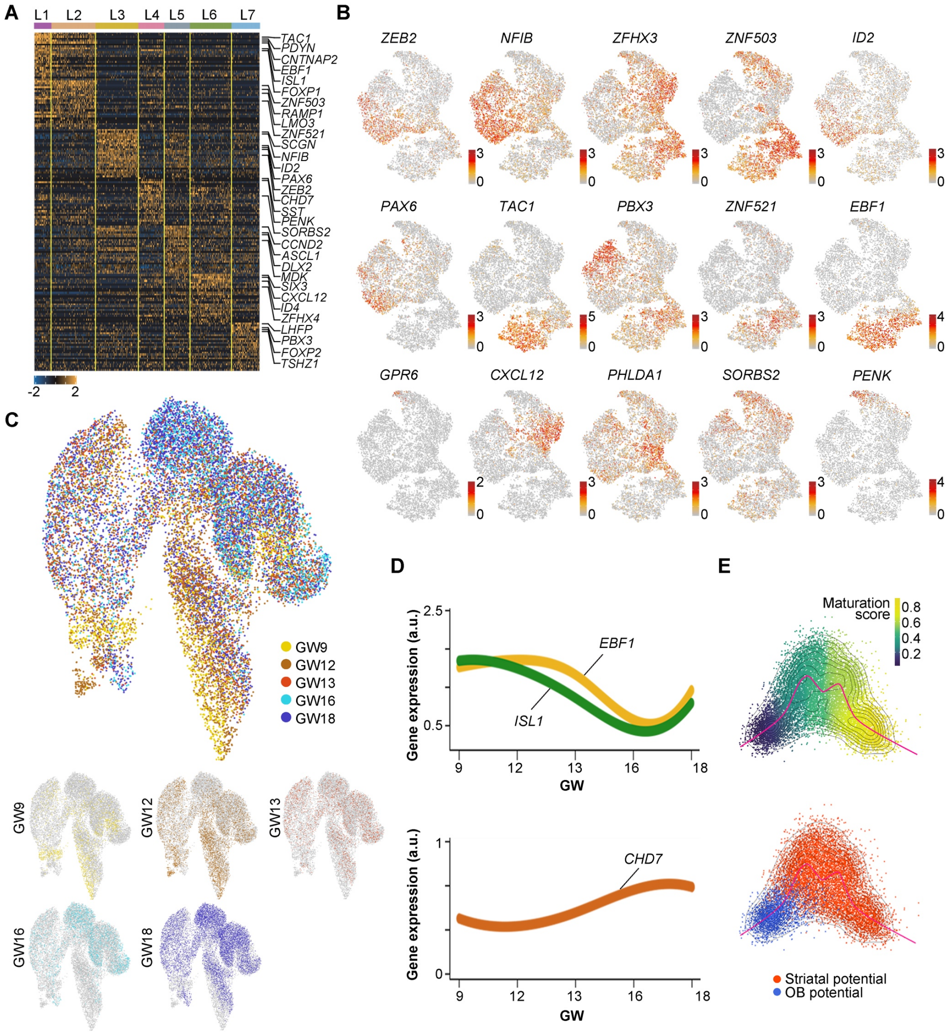

Fig. S6. Molecular regulation of early cell specification in the human LGE. (**A**) Heatmap showing the DEGs expression among subclusters of postmitotic cells in human LGE. (**B**) The gene expression in distinct subclusters of LGE postmitotic cells are visualized. Cells are colored according to the gene expression levels (red, high; gray, low). (**C**) The information of gestational weeks of human LGE cells (pLGE and postmitotic cells of LGE) is illustrated. Each dot represents a single cell and is colored by gestational weeks. (**D**) Fitted curves showing the expression pattern of *EBF1*, *ISL1* and *CHD7* in postmitotic cells of human LGE along gestational weeks. (**E**) Ordering human postmitotic LGE cells along a maturation trajectory. A principal curve was fitted to the dominant diffusion map coordinates (upper). The postmitotic LGE cells with striatal and OB potential are colored distinctly (lower).

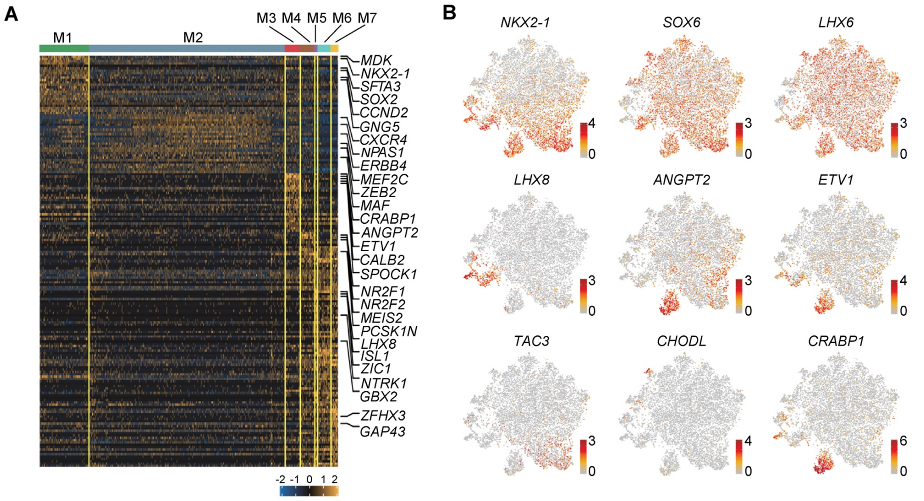

Fig. S7. The genetic regulation underlying human MGE cell differentiation.
(A) Heatmap showing the DEGs expression in subclusters of MGE post mitotic cells. (**B**) Gene expression in postmitotic cells of human MGE is visualized via t-SNE. Cells are colored according to the gene expression levels (red, high; gray, low).

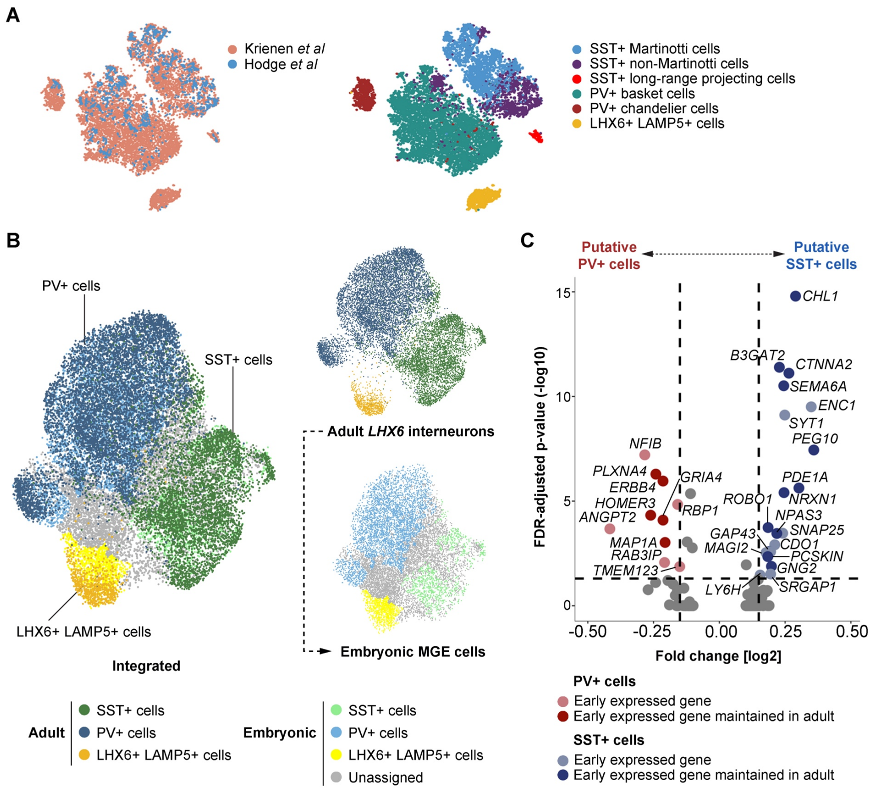

Fig. S8 Early specification of PV+ and SST+ interneurons. (**A**) The *LHX6*+ adult human cortical interneurons used in our study are derived from the datasets of Krienen *et al* and Hodge *et al*. The different types of interneuron in the integrated dataset are also shown. (**B**) Postmitotic human MGE cells (M2 and M3) and *LHX6*+ adult human cortical interneurons were integrated and visualized via UMAP. *PV*+, *SST*+ and *LHX6*+*LAMP5*+ MGE cells were annotated according to the classification of adult cortical interneurons based on transcriptional similarities. (**C**) Volcano plots depicting the differential gene expression between putative *SST*+ and *PV*+ cells in MGE.

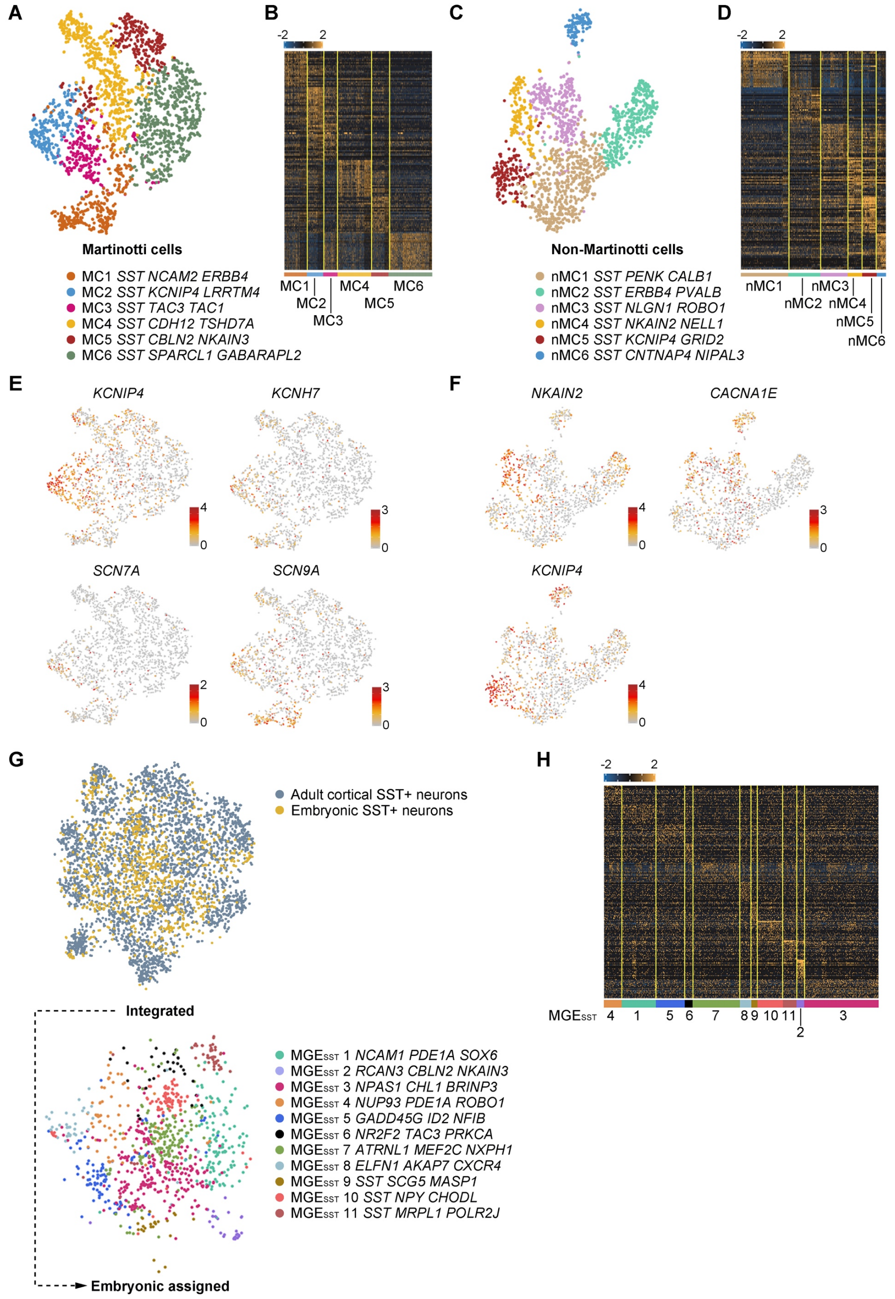

Fig. S9. Early diversification of *SST*+ interneurons. (**A**) The diversity of *SST*+ Martinotti cells in the adult cortex visualized via t-SNE. The annotation of each cell type according to gene expression is listed. (**B**) Heatmap showing the DEGs expression among different SST+ Martinotti cells. (**C**) The diversity of *SST*+ non-Martinotti cells in the adult cortex are visualized via t-SNE. The annotation of each cell type according to gene expression is listed. (**D**) Heatmap showing the DEGs expression among different *SST*+ non-Martinotti cells. (**E** and **F**) The expression pattern of genes related to functionally electrical activities is visualized in t-SNE. Cells are colored according to the gene expression levels (red, high; gray, low). (**G**) The embryonic *SST*+ cells were integrated with the adult cortical *SST*+ cells and annotated based

on transcriptional similarities to their adult counterparts. (**H**) Heatmap showing the DEGs expression in subclusters of embryonic SST+ neurons.

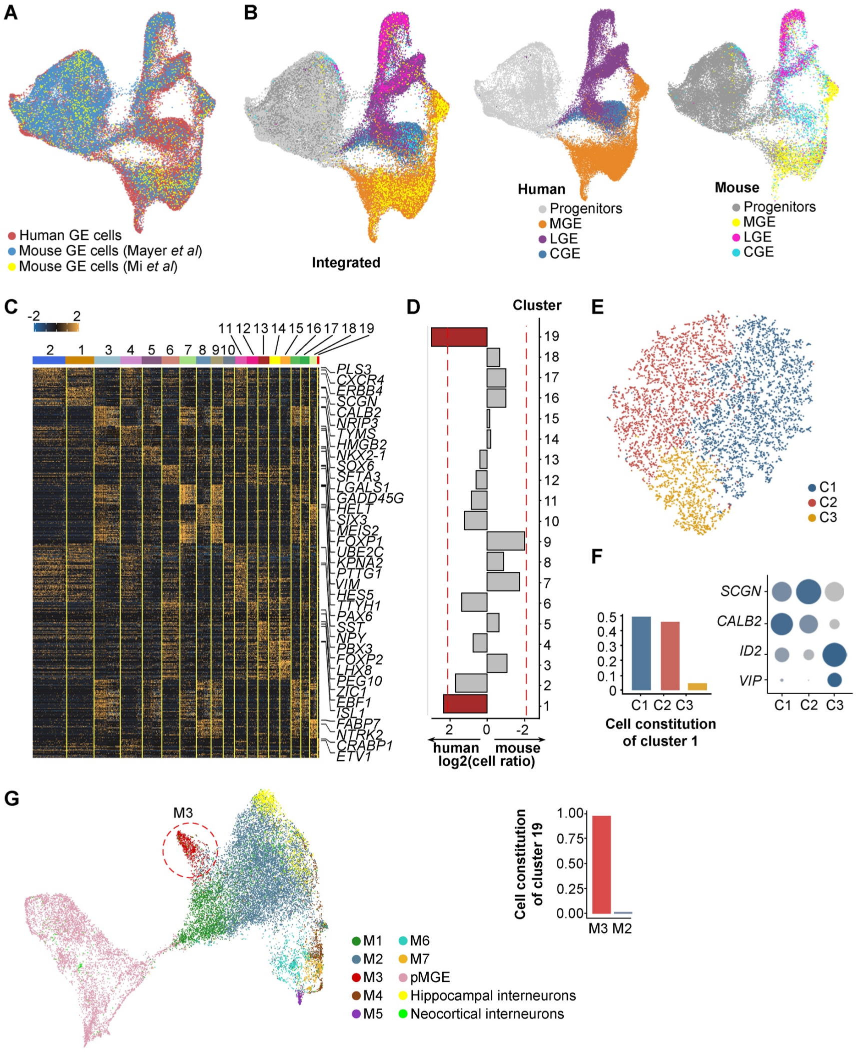

Fig. S10. Human-specific features in developing interneurons. (**A**) The cell sources of the integrated human and mouse ganglionic eminence cells was visualized by UMAP. Cells are colored according to the cell sources. (**B**) The integrated embryonic human and mouse ganglionic eminence cells are colored according to regional identity. The separated human and mouse ganglionic eminence cells are also visualized individually by UMAP. (**C**) Heatmap showing the DEGs expression among subclusters of integrated human and mouse ganglionic eminence cells. (**D**) The histogram showing the normalized cell constitution in each group. When the score of log2 (cell ratio) are beyond 2 and -2, the cell clusters are defined as human and mouse-specific, respectively. (**E**) The cell diversity of human CGE cells is visualized by t-SNE. (**F**) The cell constitution of cluster 1 is illustrated via histogram (left). The differential gene expression in subclusters of human CGE cells is also shown (right). (**G**) The cell constitution and distribution of cluster 19 are illustrated.

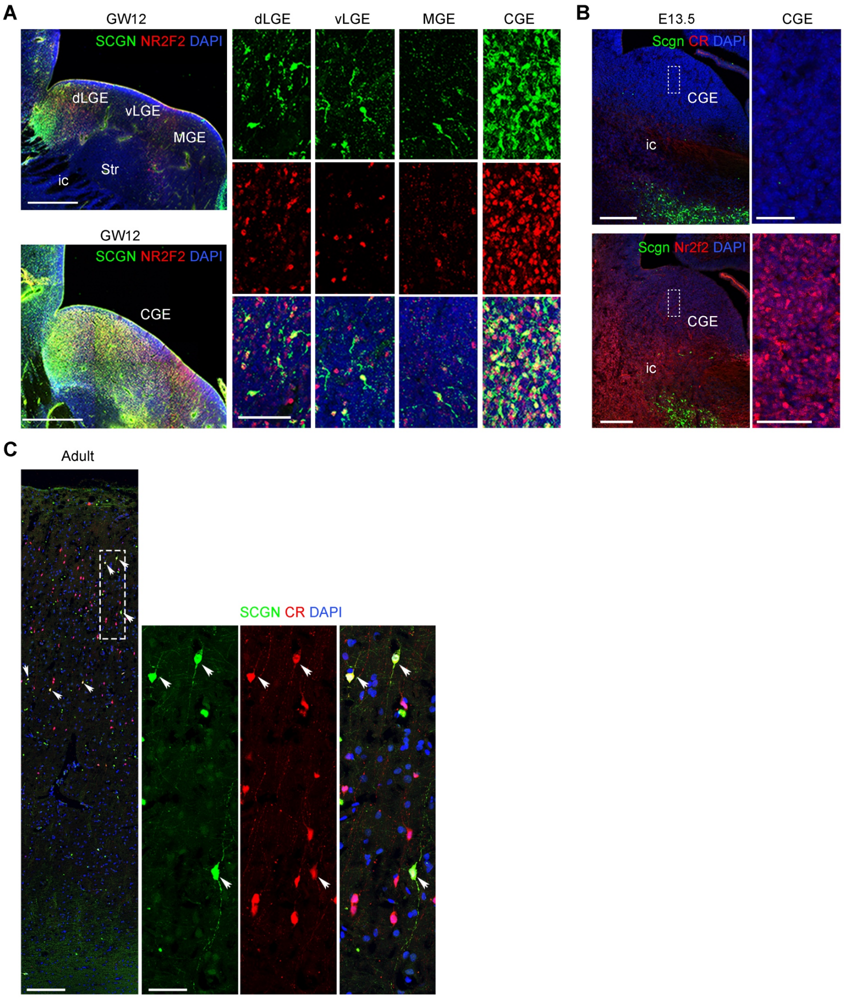

Fig. S11. Expression of SCGN and CR in the human and mouse brain. (**A**) Immunofluorescence staining for SCGN and NR2F2 in the human MGE, LGE and CGE at GW12. Scale bars, 1 mm (left), 50 μm (right). (**B**) Immunofluorescence staining for SCGN /CALB2 (upper) and SCGN/NR2F2 (lower) in the mouse CGE at E13.5. Scale bars, 200 μm (left), 25 μm (right). (**C**) Immunofluorescence staining for SCGN and CALB2 in the adult human cortex. The area in the white box is shown at high magnification. Scale bars, 200 μm (left), 50 μm (right).

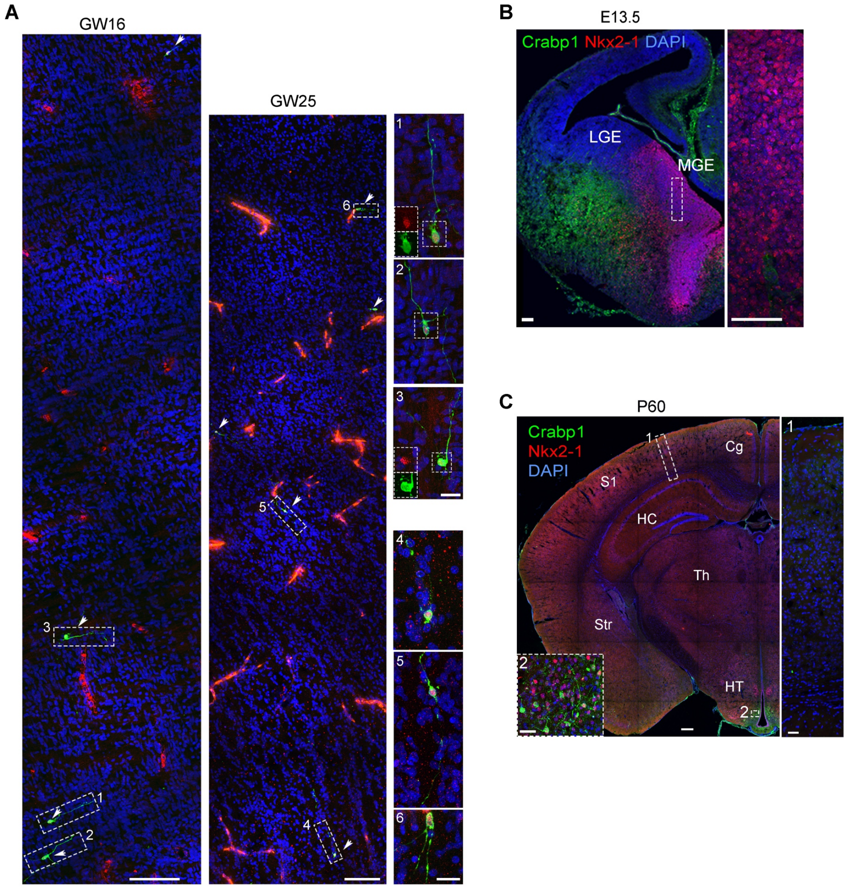

Fig. S12. Expression of CRABP1 and NKX2-1 in the human and mouse brain. (**A**) Immunofluorescence staining for CRABP1and NKX2-1 in the GW16 and GW25 human cortex. The cells outlined in the boxes are magnified to show the cell morphology. Scale bars, 100μm; for box 1-6, 20 μm. (**B**) Immunofluorescence staining for CRABP1and NKX2-1 in the mouse telencephalon at E13.5. Scale bars, 100 μm (left); 50 μm (right). **(C)** Immunofluorescence staining for CRABP1and NKX2-1 in the telencephalon of a 3-months old mouse.

Table S1. Summary of sample information.

Sample	GW09	GW12-01	GW12-02	GW13	GW16	GW18-01	GW18-02-MGE	GW18-02-LGE	GW18-02-CGE
Gender	male	male	male	male	female	female	female	female	female
Filtered cells	5257	6712	10855	5455	6187	4852	4470	6570	6054
Mean genes	1961	1596	1997	1439	1419	1234	1375	1252	1201

Table S2. Cells in Fig. 1B UMAP (separate file).

Table S3. DEGs among major cell types (separate file).

Table S4. DEGs between progenitors and postmitotic cells in the human ganglionic eminences (separate file).

Table S5. DEGs between RGCs and IPCs in the human ganglionic eminences (separate file).

Table S6. DEGs among the subclusters of ganglionic eminence progenitors (separate file).

Table S7. The genes that potentially contribute to the developmental divergence at branch point 1 and 2 of Fig.3A, respectively (separate file).

Table S8. DEGs between LGE cells with OB and striatal potential (separate file).

Table S9. DEGs among clusters of integrated dataset of human and mouse ganglionic eminence cells (separate file).

Movie S1. The spatial distribution of different clusters of human ganglionic eminence cells is displayed in 3D dimension.

References

69. G. X. Zheng *et al.*, Massively parallel digital transcriptional profiling of single cells. *Nature communications* **8**, 14049 (2017).

70. C. Trapnell *et al.*, The dynamics and regulators of cell fate decisions are revealed by pseudotemporal ordering of single cells. *Nature Biotechnology* **32**, 381-U251 (2014).

71. X. J. Qiu *et al.*, Single-cell mRNA quantification and differential analysis with Census. *Nature Methods* **14**, 309-+ (2017).

72. W. Huang da, B. T. Sherman, R. A. Lempicki, Systematic and integrative analysis of large gene lists using DAVID bioinformatics resources. *Nat Protoc* **4**, 44-57 (2009).

73. W. Huang da, B. T. Sherman, R. A. Lempicki, Bioinformatics enrichment tools: paths toward the comprehensive functional analysis of large gene lists. *Nucleic Acids Res* **37**, 1-13 (2009).

74. A. Subramanian *et al.*, Gene set enrichment analysis: a knowledge-based approach for interpreting genome-wide expression profiles. *Proc Natl Acad Sci U S A* **102**, 15545-15550 (2005).

75. V. K. Mootha *et al.*, PGC-1alpha-responsive genes involved in oxidative phosphorylation are coordinately downregulated in human diabetes. *Nat Genet* **34**, 267-273 (2003).
